# Clinical Safety and Efficacy of Allogeneic Adipose Stem Cells: A Systematic Review of the Clinical Trials

**DOI:** 10.3390/ijms26136376

**Published:** 2025-07-02

**Authors:** Alexandre Asch, Daniel F. Kalbermatten, Srinivas Madduri

**Affiliations:** 1 Division of Plastic, Reconstructive and Aesthetic Surgery, Geneva University Hospitals, 1205 Geneva, Switzerland; 2 Bioengineering and Neuroregeneration Laboratory, Department of Surgery, University of Geneva, 1205 Geneva, Switzerland

**Keywords:** allogeneic adipose stem cells, regenerative medicine, ethics

## Abstract

Allogeneic adipose stem cells (AASCs) are increasingly recognized for their potential in regenerative medicine. They offer a promising alternative to autologous cells, with potential advantages such as availability and reduced morbidity in the recipient. Therefore, the aim of the present systematic review was to explore AASCs applications in various diseases and conditions, including skin lesions, Crohn’s disease, glandular dysfunction, kidney disease, spinal muscular atrophy and osteoarthritis. This review was conducted according to PRISMA guidelines; PubMed, Embase and Web of Science databases were used to search for studies published between 2011 and 2024, without language restrictions. Our review was strictly limited to the inclusion of controlled clinical trials to ensure the relevance and quality of the data. After screening, 22 articles were retained, with a total of 953 patients that met the established inclusion criteria. The data obtained from these studies showed that AASCs have promising efficacy in improving scars and ulcers, managing Crohn’s disease, and treating glandular dysfunction and kidney disease. In spinal muscular atrophy and osteoarthritis, preliminary results also suggested potential benefits. AASCs-based treatments were well tolerated with no major adverse effects, thus emphasizing their favorable safety profile. AASCs show a significant potential for a variety of clinical applications, but the results must be interpreted with caution due to the methodological limitations of the included studies. Well-designed Phase III clinical trials are needed to confirm these promising results and ensure the safe and effective use of AASCs in clinical practice. This review highlights the importance of the standardization of procedures and raises relevant ethical issues related to the use of donor cells.

## 1. Introduction

Stem cells serve as the foundational progenitor cells in biological systems, giving rise to various differentiated cells that perform specialized functions. These undifferentiated cells possess a unique capacity for self-renewal and multilineage differentiation, which can proliferate and differentiate into diverse specialized cell types required for tissue generation, repair, and cellular homeostasis under physiologically appropriate conditions or controlled experimental environments. Under the latter conditions in the body or in the laboratory, stem cells divide to form more cells called ‘daughter’ cells. These daughter cells become either new stem cells or, after a stage called differentiation, specialized cells with a more specific function. Stem cells can be classified into the following two broad categories according to their origin. (1) Embryonic stem cells: these are totipotent stem cells, which means they can divide into more stem cells or become any type of cell in the body [1]. (2) Adult stem cells: these stem cells are found in small numbers in most adult tissues, such as bone marrow or fat. Compared to embryonic stem cells, adult stem cells have a more limited capacity to give rise to various body cells.

Currently, there are two primary methods available for repairing damaged organs or tissues in medical practice. The first method involves the complete replacement of the affected organ through transplantation. Organ transplantation typically relies on donor availability and compatibility, making it subject to limitations such as donor shortages, the risk of immune rejection, and the necessity for lifelong immunosuppressive therapy to prevent rejection and ensure successful integration. The second method consists of implanting cells into the damaged organ or tissue, a technique known as cell therapy. These cells are intended to either partially or fully restore the functionality of the affected organ. Cell therapies may use stem cells, progenitor cells, or differentiated cells depending on the specific needs and characteristics of the damaged tissue. Stem cells in particular have significant regenerative potential due to their ability to proliferate and differentiate into various cell types.

The choice between these two therapeutic strategies depends on multiple factors. These include the type and anatomical complexity of the organ, its intrinsic capacity for self-repair through resident stem cells, and the severity, nature, and extent of the damage. Organs with higher regenerative potential, such as the liver, may require less invasive methods, relying more effectively on cell therapy techniques. Conversely, organs with limited or no regenerative capability, like the heart or kidneys, might necessitate full transplantation or advanced cell therapies designed to stimulate regeneration. Additionally, the decision-making process must take into account patient-specific factors, such as age, overall health status, and potential risks or complications associated with each treatment modality [2]. With the development and application of stem cell technology, stem cell research is intensifying, and treatment is increasing rapidly each year, but there is a lack of evidence-based data on their indications. Stem cells can be used to improve health care, either by increasing the body’s regenerative potential or by developing new therapies [3], and their use in medical research may lead to the discovery of new ways of treating currently incurable diseases.

**Figure 1:**
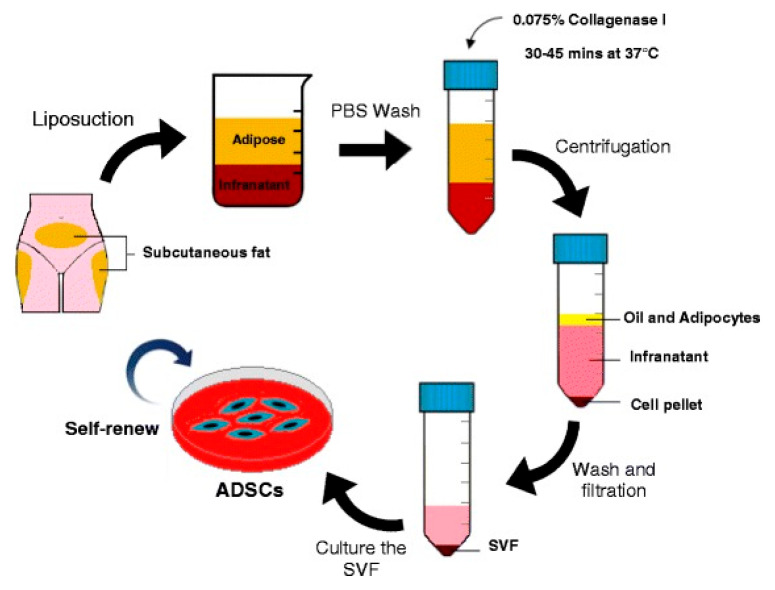
The process to extract AASCs from human fat obtained by liposuction begins with thoroughly cleansing the lipoaspirate in Phosphate buffered saline to eliminate any blood or impurities. Next, the fat is broken down enzymatically to separate out the stromal vascular fraction (SVF), which is then purified using filtration and centrifugal techniques. Cultivating the SVF in regular plastic flasks used for tissue culture encourages the selective proliferation of the adipose stem cell population. (Reprinted without modifications from Ciervo et al. [12]).

Allogeneic adipose stem cells (AASCs) represent a fascinating field of biomedical research and have attracted growing interest due to their diverse therapeutic potential. These cells derived from adipose tissue open up new perspectives in the field of regenerative medicine and offer innovative solutions for various diseases affecting the digestive system, skin, eyes, kidneys, muscles or even bones [4] (Figure 1).

AASCs, also known as adipose tissue-derived mesenchymal stem cells, are extracted from the adipose tissue of various donors. These cells have a unique plasticity that enables them to differentiate into various cell types, including bone, cartilage and adipose cells [5]. Allogeneic adipose-derived stromal cells (AASCs) have emerged as a particularly promising source for regenerative therapies, offering distinct advantages over autologous counterparts. One of the key benefits is their immediate availability, which eliminates the need for patient-specific harvesting procedures. This not only spares patients invasive and often painful tissue collection but also allows for rapid clinical intervention, a crucial advantage in acute or time-sensitive indications [4]. Unlike autologous cells, which are subject to patient-related variability in cell yield and quality, particularly in older adult or comorbid populations, AASCs are obtained from healthy, rigorously screened donors.

These donor-derived cells can be expanded in controlled environments to produce homogeneous, well-characterized cell populations with reproducible therapeutic properties. Such standardization is critical for ensuring consistency, safety and regulatory compliance across clinical applications [5]. Biologically, AASCs possess a favorable immunological profile. Their low expression of HLA class II molecules, coupled with a lack of co-stimulatory markers and their secretion of anti-inflammatory cytokines, underlies their immunomodulatory properties. These features reduce the likelihood of triggering a strong host immune response, even when administered in an allogeneic context [4,5].

AASCs are also a rich source of bioactive molecules that contribute to tissue repair through paracrine mechanisms. In vitro studies have demonstrated their secretion of a wide range of growth factors, including platelet-derived growth factor (PDGF), vascular endothelial growth factor (VEGF), transforming growth factor-β (TGF-β), and insulin-like growth factor (IGF), all of which modulate angiogenesis, stimulate cell proliferation, and support extracellular matrix remodeling [3].

These biomolecules are essential for modulating angiogenesis, stimulating cell proliferation and promoting extracellular matrix, thus contributing to the architecture and function of repaired tissue [6]. For instance, VEGF plays a central role in promoting neovascularization and oxygen delivery to healing tissue [7], while PDGF supports progenitor cell recruitment and survival [8].

Across regenerative-medicine applications, AASCs are increasingly combined with tailor-made biomaterials to boost both safety and efficacy. In cutaneous repair and burn treatment, AASCs seeded into natural hydrogels (collagen, fibrin or hyaluronic acid) accelerate re-epithelialization and attenuate inflammation; in bone and cartilage defects, their osteo- and chondro-genic potential is harnessed within mineralized foams or 3D-printed composite scaffolds; and in soft-tissue augmentation or fistula closure, injectable matrices or thin films provide immediate structural support while protecting cells from mechanical stress. Beyond simply hosting the cells, these carriers can be engineered to modulate degradation kinetics, deliver adjuvant cytokines or present topographical cues, thereby prolonging cell viability and synchronizing growth-factor release with the needs of the host tissue [9].

While these effects are well-established in vitro, emerging in vivo data also suggest that AASCs may serve as a transient but functionally significant source of growth factors post-implantation. For example, in a recent study, AASCs embedded in hyaluronic acid-based scaffolds continued to secrete VEGF and TGF-β for up to seven days after implantation in rat models, as measured by ELISA [9]. Similarly, implantation of AASCs in rabbit models of cartilage injury demonstrated expression of chondrogenic markers and signs of paracrine-driven tissue modulation up to twelve weeks post-implantation [10]

**Figure 2:**
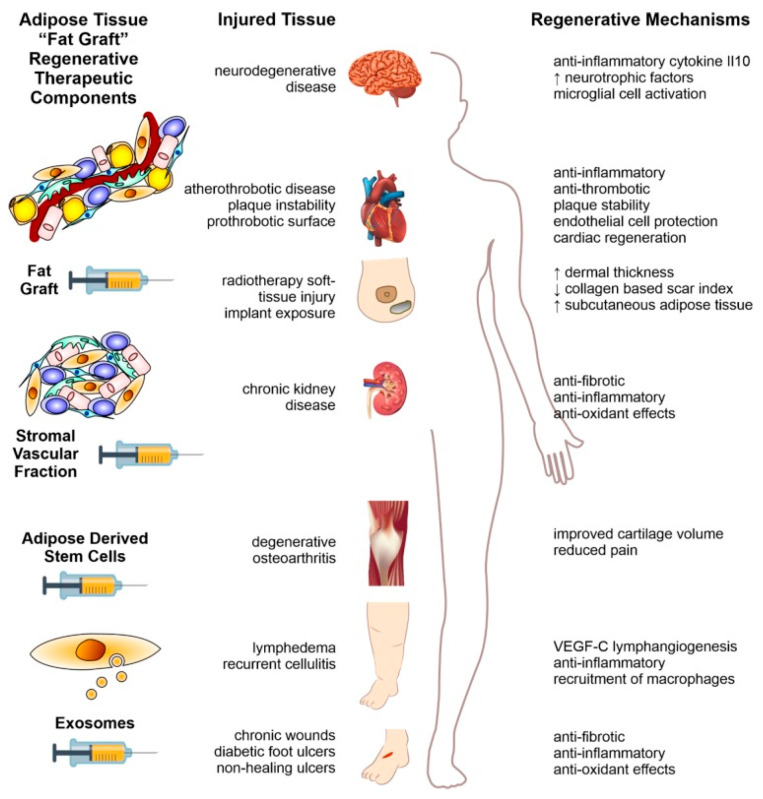
Treatment approaches using fat from adipose tissue for human illnesses are outlined schematically. This involves a step-by-step refinement of a basic fat graft collected via liposuction from beneath the skin. Starting from the initial fat graft, the process includes enzymatic processing to derive the stromal vascular fraction pellet, and then further processing to isolate AASCs and extracellular vesicles (as indicated in the left column of the summary). Each step in this refinement shows considerable promise for the therapeutic reversal of various diseases affecting multiple body systems, as detailed in the middle column of the summary. The right column depicts the processes through which these therapeutic effects are achieved. (Reprinted without modifications from Shukla et al. [13]).

Therefore, the clinical application of AASCs for tissue reconstruction is supported not only by their differentiation potential but also by their active, though possibly transient, paracrine signaling in vivo, which plays a critical role in orchestrating tissue repair processes [11] (Figure 2).

This review is intended to be exhaustive, with the aim of providing a brief overview of the past, present and future of AASC therapies in clinical practice.

## 2. Materials and Methods

### 2.1. Search STRATEGY

This review was conducted according to PRISMA (Preferred Reporting Items for Systematic Reviews and Meta-Analyses) guidelines. PubMed, Embase and Web of Science databases were used to search for studies published between January 2011 and October 2024 relating to the use of AASCs, with no language restrictions. This time frame was selected to ensure the inclusion of the most recent and relevant data available, thereby maximizing the precision and contemporary relevance of the review.

To ensure both sensitivity and reproducibility, the search strategy combined controlled vocabulary, such as MeSH and Emtree terms, with relevant free-text keywords and Boolean operators (AND, OR, NOT) to optimize the sensitivity and specificity of the search. Terms such as “allogeneic stem cells”, “adipose-derived stem cells”, “mesenchymal stem cells”, “clinical trials”, “clinical applications” and “donor-derived” were included, with variations adapted specifically to the indexing systems of each database to maximize coverage and retrieval accuracy. For example, a representative query was formulated as follows: ((“adipose-derived stem cells” OR “mesenchymal stem cells” OR “allogeneic stem cells”) AND (“clinical trials” OR “clinical applications” OR “therapy”) AND (“donor-derived” OR “allogeneic” OR “off-the-shelf”) NOT “autologous”). This search string incorporated all core concepts and terminologies relevant to the review, including variations of stem cell types (“adipose-derived”, “mesenchymal”, “allogeneic”), clinical context (“clinical trials”, “clinical applications”, “therapy”) and graft origin (“donor-derived”, “off-the-shelf”), while actively excluding studies focused solely on autologous cells. Boolean operators (AND, OR, NOT), parentheses and truncation were applied systematically to structure the logic of the search. Queries were further adapted to each database using both controlled vocabulary (e.g., MeSH, Emtree) and free-text keywords, ensuring comprehensive, specific and reproducible retrieval of relevant studies.

### 2.2. Inclusion and Exclusion Criteria

Studies were selected based on clearly defined inclusion and exclusion criteria to ensure methodological rigor and clinical relevance. The review was strictly limited to controlled clinical trials, defined as prospective interventional studies involving human participants in which the effects of AASCs were evaluated against a control group, such as placebo, standard care, or an alternative intervention. The majority of included studies were published between 2022 and 2024, in line with our aim to emphasize the most recent and clinically applicable findings.

Study eligibility was anchored in a priori PICOS criteria to ensure both methodological rigor and clinical relevance. Eligible populations included human participants of any age who were treated for a clinical condition in which allogeneic adipose-derived stem cells were investigated. The intervention of interest was the administration of AASCs, delivered either as a standalone therapy or in combination with another regimen. Acceptable comparators comprised placebo, standard care, or an active alternative intervention. Prespecified outcomes encompassed efficacy end points, such as functional or disease-specific scores and safety end points, notably adverse and serious adverse events. Finally, only study designs that met the definition of prospective, controlled clinical trials were included.

The selection process was conducted in two stages: an initial screening of titles and abstracts, followed by a full-text review to confirm eligibility. Inclusion criteria were applied to studies presenting original data specifically related to the therapeutic use of AASCs. Exclusion criteria included review articles, meta-analyses, background literature and studies deemed unrelated to the topic.

### 2.3. Extraction of Relevant Data, Quality, and Risk of Bias Assessment

Two reviewers independently populated a piloted data-extraction form that captured, for every trial, (i) study design (randomized vs. non-randomized; parallel, crossover or cluster), (ii) sample size analyzed, (iii) clinical indication for AASC therapy, (iv) details of the intervention and comparator, (v) length of follow-up and (vi) all prespecified efficacy and safety outcomes with their numerical results (point estimate, measure of dispersion and time point).

Methodological quality was then appraised with a formal, design-specific risk-of-bias instrument: RoB 2.0 for randomized controlled trials and ROBINS-I for non-randomized controlled trials.

RoB 2.0 judges five domains (randomization process, deviations from intended interventions, missing outcome data, outcome measurement and selection of the reported result).

ROBINS-I judges seven domains (confounding, participant selection, intervention classification, deviations from intended interventions, missing data, outcome measurement and selection of the reported result).

For each methodological domain assessed, we assigned a risk level categorized as low, moderate or high, based on predefined criteria. These individual assessments were then systematically integrated to determine an overall risk of bias rating for each included study, providing a comprehensive evaluation of its methodological quality.

All disagreements, whether in data extraction or risk-of-bias judgement, were resolved by consensus (Table 1).

## 3. Results

**Figure 3:**
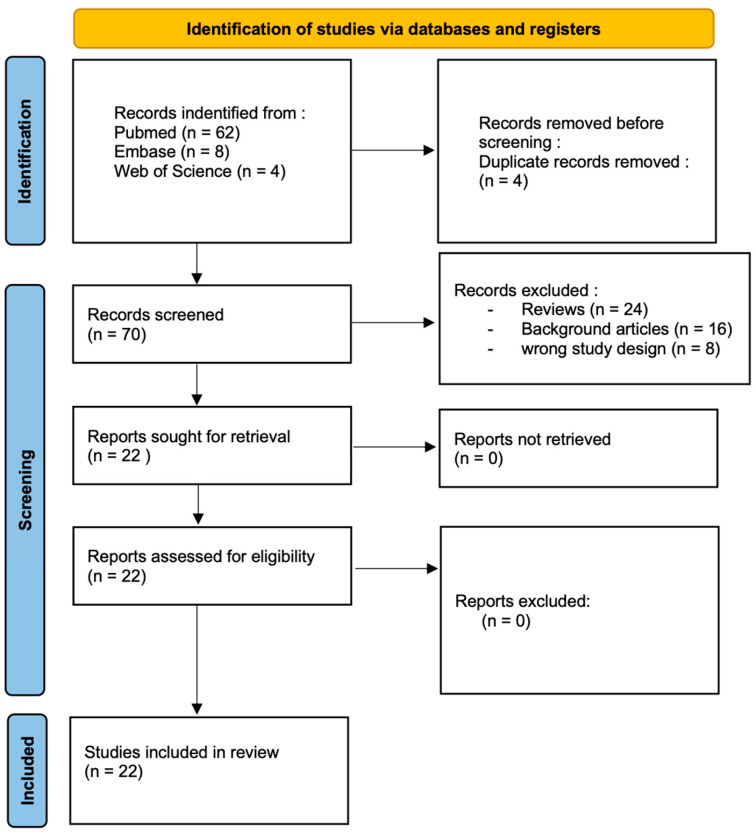
The PRISMA flow chart describing the process of the article’s selection for the present systematic review. In the preliminary search, 62 articles on PubMed, eight articles on Embase and four articles on Web of Science were found.

From a total of 74 research papers, 70 articles were selected after duplicates were removed (*n* = 4). Of these 70 papers, after title and abstract evaluation by two independent researchers, 24 studies were excluded because they were reviews, 16 were excluded because they were background articles, and eight were excluded because they did not use the correct study design (Figure 3).

Following this screening, a total of 22 studies were included in the final review, covering a total of 953 patients.

Methodological quality was appraised with the Cochrane RoB 2 framework for randomized trials (nine studies) and ROBINS-I for non-randomized designs (13 studies). Agreement between assessors was excellent. Seven studies (32%) satisfied all critical standards and were judged at low risk of bias. Nine (41%) were rated moderate, most often because allocation concealment was insufficiently described or secondary outcomes were selectively reported. The remaining six (27%) were deemed high risk, typically owing to open-label conduct without blinded outcome assessment or attrition exceeding 20%.

This structured and transparent risk-of-bias assessment ensured a robust appraisal of methodological quality across study designs, thereby strengthening the interpretability and credibility of the review’s findings.

### 3.1. Overview of the Efficacy of AASC Treatment

Skin Lesions: Across four studies involving 190 patients, 103 (54.2%) received AASCs. Among those treated, 93 patients (90.3%) benefited from the treatment, while only 10 patients (5.3% of the total population studied) showed no improvement.

Crohn’s Disease: Three studies included 271 patients, with 151 (55.72%) receiving AASCs. Of these, 88 patients (58.28% of those treated) benefited from the treatment, whereas 63 patients (23.25% of the total population) experienced no improvement.

Glandular Dysfunction: Four studies involved 197 patients, of whom 102 (50%) received AASCs. Half of these treated patients (51, or 50%) showed improvements, while the other 51 patients (25.89% of the total population) received no benefit.

Lung Diseases: In three studies involving 172 patients, 90 (52.33%) received AASCs, with 62 patients (68.89% of those treated) benefiting from the treatment. Conversely, 28 patients (16.28% of the total population) did not experience any improvement.

Strokes: A single study included 13 patients, four of whom (30.77%) received AASCs. Notably, all four patients (100% of those treated) showed significant benefits from the treatment.

Renal Dysfunction: In a single study of 12 patients, all received AASCs. Among them, six patients (50%) benefited from the treatment.

Neurodegenerative Diseases: A single study treated 10 patients, including five (50% of the total population) who received AASCs. Remarkably, all five patients (100% of those treated) benefited from the treatment.

Osteoarthritis: Four studies involving 76 patients found that 56 patients (73.68%) received AASCs. Among those treated, 50 patients (89.29%) benefited from the treatment, while six patients (7.89% of the total population) showed no improvement.

Acute Graft-Versus-Host Disease (GvHD): A study of 12 patients, all of whom received AASCs, reported that nine patients (75%) benefited from the treatment.

Overall Results: Across 953 patients analyzed in these studies, 535 patients (56.14%) received AASCs. Among those treated, 368 patients (68.79%) experienced significant benefits, while 167 patients (17.52% of the total population) showed no improvement (Table 2).

### 3.2. Skin Lesions

#### 3.2.1. A Scar Treatment

According to Gentil [14], 50 patients with scars and soft tissue deformities with hyperpigmentation treated with fat grafting, commonly known as “lipofilling”, enhanced with adipose tissue-derived mesenchymal stem cells (lipofilling-AASCs) and 50 patients treated with unenhanced lipofilling (lipofilling-NE) were prospectively evaluated. Preoperative evaluation included clinical assessment, photographic assessment, magnetic resonance imaging (MRI) and ultrasound. Postoperative follow-up was performed at weeks 1, 3, 7, 12, 24 and 48, then annually. Improvement in volume contours and pigmentation was clinically evaluated.

All those who underwent treatment (lipofilling-AASCs and lipofilling-NE) were satisfied with the improvement in pigmentation, texture and volume contours, with some differences. In the first subpopulation, one year after lipofilling-AASCs treatment, 25 patients were very satisfied (50%), 19 were satisfied (38%), and six patients were dissatisfied (12%). In the second subpopulation, 1 year after lipofilling-NE, 10 patients were very satisfied (20%),18 patients were satisfied (36%), and 22 patients were dissatisfied (44%). In conclusion, lipofilling-AASCs was the preferred option for improving contour deformities associated with increased scar pigmentation.

#### 3.2.2. Treatment of Ulcers

Moon et al. [15] conducted a clinical trial to evaluate the efficacy of a treatment based on adipose tissue-derived stem cells in 39 patients with diabetic foot ulcers. Inclusion criteria included age 18–80 years, type 1 or 2 diabetes, ulcer history of more than 4 weeks, wound size 1–25 cm^2^, and a Wagner wound depth grade 1 or 2. Additional criteria included detectable blood flow, an ankle brachial index between >0.7 and <1.3, and transcutaneous oxygen pressure > 30 mmHg. Exclusion criteria included wound size changes of >30% in one week, wound infection, HIV status, HbA1c > 15% and postprandial glucose > 450 mg/dL. Patients were randomized into two treatment groups.

At week 8, 73% of subjects in the treatment group (16 of 22) achieved complete wound closure, compared with 47% in the control group (8 of 17). For secondary endpoints, at week 12, 82% of subjects in the treatment group achieved complete closure (18 of 22) versus 53% in the control group (9 of 17), with a trend towards significance (*p* = 0.053). Mean time to complete healing was 40.8 ± 5.3 days in the treatment group and 51.2 ± 3.9 days in the control group. According to the Kaplan–Meier median, the treatment group had a significantly shorter time to complete healing (28.5 days) than the control group (63.0 days) (*p* = 0.033).

A study published by Mrozikiewicz-Rakowska et al. [16] on this topic involved two equivalent groups of 23 participants receiving either fibrin gel with AASCs or fibrin gel alone. Clinical assessment was performed at four different time points: days 7, 14, 21 and 49. Wound size reduction was significantly greater on days 21 and 49 in patients receiving AASCs. Time to a 50% reduction in wound size in the fibrin gel group was 25.5 ± 4.2 days and 17.6 ± 1.5 days in the AASCs group (*p* = 0.029).

**Figure 4:**
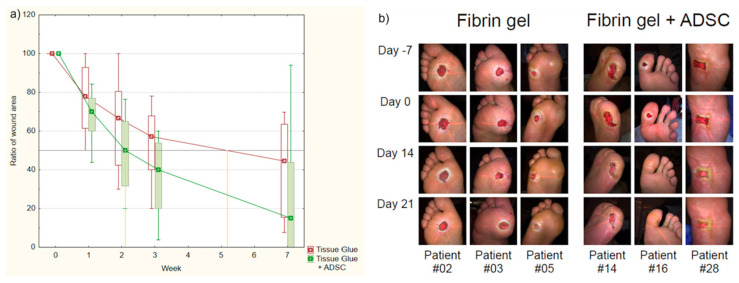
Comparison of treatment progress between patients in the fibrin gel group and AASC group. (**a**) Tracking the change in wound size over time in both groups. The relative wound size is calculated as the ratio of the wound size at a given week to its size at week 0. Patients initially received either tissue glue alone or tissue glue in combination with AASCs. (**b**) Visual documentation of wound areas in the examined groups, illustrating the progression of treatment for diabetic foot ulcer and highlighting differences between patients in the fibrin gel and AASCs groups. Photos were taken weekly, starting from the screening visit (day 7), treatment application (day 0), and at 2 and t3 weeks into treatment (days 14 and 21, respectively). (Reprinted without modifications from Mrozikiewicz-Rakowska et al. [16]).

Seven patients treated with AASCs achieved complete healing at the end of the study compared with one patient in the non-AASCs group. One week after the application of AASCs, 34 proteins differed significantly between groups, seven of which were positively correlated with the healing rate, including GAPDH, CAT, ACTN1, KRT1, KRT9, SCL4A1 and TPI. These results confirmed the improved wound healing associated with AASCs, thus offering molecular insights and contributing to the understanding of the role of AASCs in wound healing (Figure 4).

#### 3.2.3. C Psoriasis Treatment

A study on the topic of psoriasis treatment by Bajouri et al. [17] involved five patients (three men and two women with a mean age of 32.8 ± 8.18 years. Three patients received a total of 3.10^6^ cells/cm^2^ of AASCs, while two patients received only 10^6^ of these cells. The latter were injected into the subcutaneous tissue of each plaque in a single dose, and changes in clinical and histological indices, the number of B and T lymphocytes in local and peripheral blood, and serum levels of inflammatory cytokines were assessed.

No major adverse effects, such as burning, pain, itching or systemic side effects, were observed after the AASC injection, and lesions showed mild-to-considerable improvement after injection. The mRNA expression levels of pro-inflammatory factors were reduced in the dermis of patients after injection. Increased expression of the transcription factor Foxp3 in patients’ blood samples suggests modulation of inflammation after AASC administration.

Six months later, skin thickness, erythema and plaque desquamation, as well as the PASI score, decreased in most patients, according to clinical analysis by the physicians in charge. This study therefore suggests that ASSC injection can be considered as a safe and effective therapeutic approach for psoriatic plaques.

#### 3.2.4. Crohn’s Disease

A study by Panés et al. [18] on Crohn’s disease involved a total of 212 patients, 107 of whom were treated with AASCs. The results revealed that 53 patients derived significant benefit from this intervention, underlining the potential efficacy of AASCs in the management of their medical condition. This was a randomized, double-blind, parallel-group, placebo-controlled study conducted from 6 July 2012 to 27 July 2015, in 49 hospitals in seven European countries and Israel.

Adult patients (≥18 years of age) with Crohn’s disease and treatment-refractory draining complex perianal fistulas were randomly assigned using a pre-established randomization list to a single intralesional injection of 120 million AASCs (Cx601) to 24 mL saline (placebo). Treatment was administered by an unmasked surgeon, a gastroenterologist and a masked radiologist assessing the therapeutic effect. The primary endpoint was a combined remission at week 24 (i.e., clinical assessment of closure of all treated external orifices draining at baseline and the absence of >2 cm collections from treated perianal fistulae, confirmed by masked central magnetic resonance [MRI] imaging).

A total of 212 patients were randomized: 107 to Cx601 and 105 to placebo. A significantly higher proportion of patients treated with Cx601 versus placebo achieved combined remission in the intention-to-treat (ITT) populations (53 out of 107 [50%] vs. 36 out of 105 [34%]; a difference of 15.2% (97.5% confidence interval [CI] 0.2–30.3; *p* = 0.024)). A total of 18 of 103 patients in the Cx601 group (17%) vs. 30 (29%) of 103 patients in the placebo group experienced treatment-related adverse events, the most frequent being anal abscess (six in the Cx601 group versus nine in the placebo group) and proctalgia (five vs. nine). In conclusion, Cx601 is an effective and safe treatment for complex perianal fistulas in Crohn’s disease patients who have failed to respond to conventional and/or biologic therapies.

An ADMIRE-CD study conducted by Garcia-Olmo et al. [19] evaluated the efficacy and safety of darvadstrocel (AASCs) for the treatment of complex perianal fistulas in Crohn’s disease over a 104-week period. Of the 131 patients who completed the initial 52-week follow-up, 40 participated in the extended follow-up, with 25 receiving darvadstrocel and 15 receiving a placebo. Patients in the darvadstrocel group had a mean age of 38.6 years compared to 42.7 years in the placebo group. The proportion of men was similar between the groups (56% for darvadstrocel vs. 53% for placebo). However, 60% of patients in the darvadstrocel group presented with more complex fistulas (more than one internal or external opening) compared to 33% in the placebo group.

During the 104-week period, four serious adverse events (SAEs) were reported: three in the darvadstrocel group (12%) and one in the placebo group (6.7%). None were attributed to the treatment. Events reported in the darvadstrocel group included anal abscess, anal fistula and fistula discharge, while fistula discharge was reported in the placebo group. These results showed a reduction in treatment-emergent serious adverse events (TESAEs) in the darvadstrocel group, decreasing from 24% at 52 weeks to 12% at 104 weeks.

**Figure 5:**
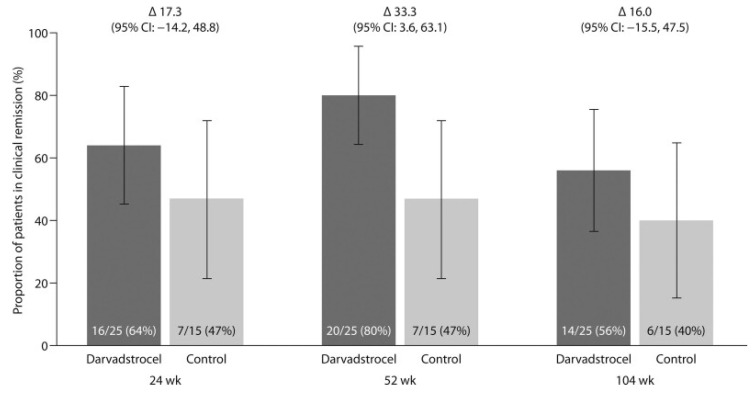
Proportion of patients achieving clinical remission at weeks 24, 52 and 104 following fistula tract curettage and administration of darvadstrocel or placebo (control group). (Reprinted without modifications from Garcia-Olmo et al. [19]).

Clinical remission, defined as the closure of all treated external openings without drainage, was achieved by 64% of patients in the darvadstrocel group compared to 47% in the placebo group at 24 weeks. At 52 weeks, clinical remission reached 80% in the darvadstrocel group versus 47% in the placebo group, representing a difference of 33% (95% CI, 3.6 to 63.1). At 104 weeks, clinical remission remained superior in the darvadstrocel group at 56%, compared to 40% for the placebo. Among patients on concomitant anti-TNF therapy, 59% of those in the darvadstrocel group were in remission at 104 weeks, compared to 30% for the placebo. For patients not receiving anti-TNF therapy, remission was 50% with darvadstrocel and 60% with the placebo (Figure 5).

In summary, darvadstrocel achieved significant clinical remission rates, reaching 80% at 52 weeks and remaining high at 56% at 104 weeks. The safety profile was comparable to the placebo, with no treatment-related serious adverse events. These results highlight the potential of darvadstrocel to provide durable remission of complex perianal fistulas in patients with Crohn’s disease, representing a significant advance in managing this challenging condition.

A study by Maciel et al. [20] aimed to evaluate the safety of allogeneic mesenchymal stem cells (MSCs) in the treatment of complex anal fistulas in patients without Crohn’s disease.

Conducted as a prospective, non-randomized phase I clinical trial in a secondary hospital, it included 20 consecutive patients diagnosed with complex anal fistulas. Each patient received a total of 40 × 10^6^ allogeneic MSCs, with 20 × 10^6^ cells applied per fistula tract in cases with two tracts.

Patients were hospitalized for 24 h post-procedure and evaluated at weeks 1, 2, 4, 8, 16 and 24, with long-term follow-up conducted one year after treatment. The intervention was performed on 20 patients between 1 October 2016 and 31 October 2017, though one patient was excluded from the final analysis.

No adverse effects were reported within the first 24 h, and all patients were discharged without symptoms. Three patients (15%) developed perianal abscesses—one at week 4 and two at week 8. Complete fistula closure was achieved in 13 patients (69%).

Although this study had the limitation of being non-randomized, it demonstrated that the use of allogeneic MSCs is a safe option for treating complex anal fistulas not associated with Crohn’s disease.

#### 3.2.5. Conclusions

Taken together, dermatologic trials show high clinical response, yet they rely on small sample sizes, inconsistent comparators and mostly non-blinded designs. The promising safety–efficacy profile therefore warrants larger, rigorously blinded studies before routine clinical adoption.

### 3.3. Glandular Dysfunctions

#### 3.3.1. Treatment of Sjogren’s Disease

**Figure 6:**
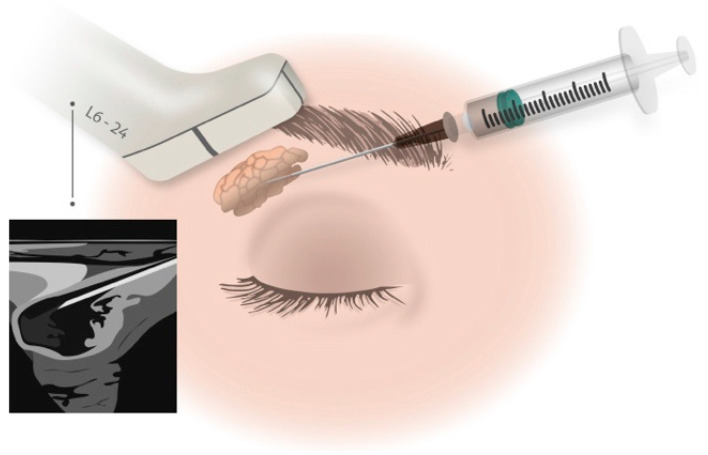
Administering an injection into the lacrimal gland with the assistance of ultrasonic guidance. (Reprinted without modifications from Moller-Hansen et al. [21]).

The study by Moller-Hansen et al. [21] included 54 participants with severe dry eye disease secondary to Sjögren’s disease and divided them into three groups, i.e., those using AASCs (n = 20), an active comparator (n = 20), or a non-randomized observation group (n = 14). The intervention groups received a single injection of AASCs or an active comparator into the lacrimal gland of one eye, while the observation group received lubricating eye drops only (Figure 6). The primary endpoint was change in the Ocular Surface Disease Index (OSDI) score, and the secondary endpoints were non-invasive tear break-up time, tear meniscus height, the Schirmer test and the Oxford score during a 12-month follow-up.

At baseline, the 54 study participants had a median OSDI score of 46.7 (interquartile range [IQR] 36.1–56.8), with no significant difference between the three groups. In the AASCs group, the OSDI score decreased significantly from the median of 39.8 at baseline by a mean of 16.6 points (−41.6%; *p* < 0.000) at 1-week follow-up, which was maintained at the 12-month follow-up (−16.1 points, −40.4%; *p* < 0.000). In the vehicle-treated group, the median OSDI score was 49.0 at baseline and decreased by a mean of 21.2 (−43.2%; *p* < 0.000) at 1-week post-treatment, which was also maintained at 12-months post-treatment (−20.8, −42.4%; *p* < 0.000).

In the observation group, the OSDI score did not change significantly during the 12-month follow-up. During the follow-up period, no significant difference was observed in the decrease of the OSDI score between the AASCs group and the vehicle group, while both interventions showed a significant decrease compared to the observation group (*p* = 0.03 and *p* = 0.004 for AASCs and vehicle, respectively).

The median Schirmer test score in the study eye was 3 mm (IQR 1–5 mm), with no difference between groups (*p* = 0.23). After 4 months’ follow-up, there was a significant mean increase in the Schirmer test score of 3.55 mm (125%; *p* = 0.01) in the AASCs group and 3.8 mm (115%; *p* = 0.008) in the vehicle group, which was maintained after the 12-month follow-up. Median tear osmolarity in the study eye at baseline was 314 mosm/L, with no difference between groups. In the AASCs group, a significant mean decrease in tear osmolarity was observed after the 12-month follow-up of 12.38 mosm/L (−3.9%; *p* < 0.05), with a trend towards a significant difference from the vehicle group after 4 months (*p* = 0.07) and at the 12-month follow-up (*p* = 0.098). An improvement in the subjective and objective signs and symptoms of this pathology was observed in both intervention groups after injection into the lacrimal gland, compared with the observation group.

#### 3.3.2. Treatment of Xerostomia

Lynggaard et al. [22] attempted to provide proof of concept of the efficacy and safety of AASCs injected into the major salivary glands of irradiated patients. Eligible patients with objective and subjective evidence of radiation-induced salivary gland damage after treatment of stage I-II oropharyngeal squamous cell carcinoma (UICC 8) were enrolled. In total, 25 million cryopreserved AASCs were injected into each submandibular gland and 50 million AASCs into each parotid gland. Data were analyzed using repeated measures linear mixed models.

A total of 10 patients (seven men, three women; 59.5 years [range: 45–70]) were treated in four glands. No treatment-related serious adverse events occurred. Over 4 months, unstimulated saliva flow increased from 0.13 mL/mi at baseline to 0.18 mL/min, a change of 0.06 (*p* = 0.0009) mL/min. Stimulated saliva flow increased from 0.66 mL/min at baseline to 0.75 mL/min, a change of 0.09 (*p* = 0.017) mL/min.

**Figure 7:**
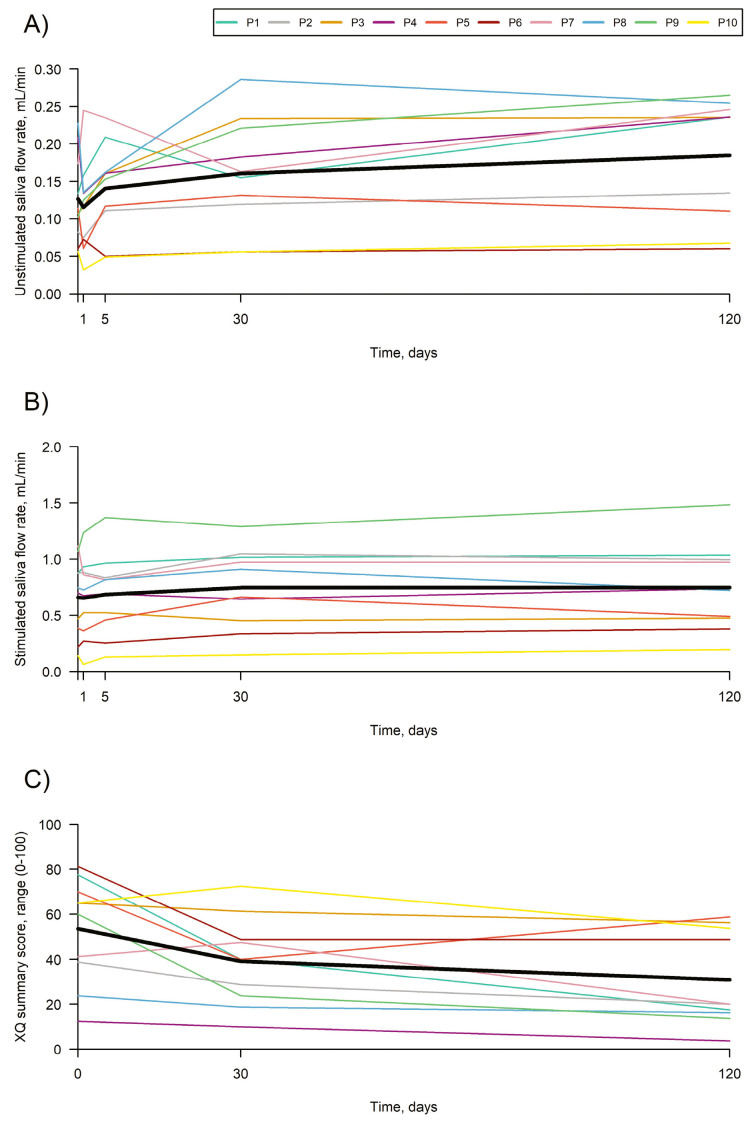
Patients with radiation-induced xerostomia. (**A**) Evolution of unstimulated whole saliva flow rate. (**B**) Changes in stimulated whole saliva flow rate. (**C**) Trend of xerostomia questionnaire summary score over time. Colored lines depict the 10 patients, and black lines represent the least square means. (Reprinted without modifications from Lynggaard et al. [22]).

The xerostomia questionnaire summary score decreased by 22.6 units (*p* = 0.0004), the European Organization for Research and Treatment of Cancer Quality of Life Questionnaire Head and Neck Module (EORTC QLQ-H&N35) (dry mouth domains decreased by 26.7 (*p* = 0.0013), sticky saliva by 23.3 (*p* = 0.0015) and swallowing by 10.0 (*p* = 0.0016). This suggests that treatment of the major salivary glands with AASCs could be a safe option (Figure 7).

Jakobsen et al. [23] enrolled 120 patients treated with AASCs an increase in unstimulated whole salivary flow rate (UWS) by 0.04 mL/min (38%) over four months, while the placebo group exhibited a smaller, non-significant increase of 0.01 mL/min (21%). In total, 13 patients (21.7%) in the AASCs group reached normal salivary flow rates (≥0.26 mL/min), compared to only eight patients (13.3%) in the placebo group. Despite these improvements, the difference in salivary function between the two groups was not statistically significant (*p* = 0.11).

For the secondary endpoints, no significant improvement in stimulated salivary flow rate (SWS) was observed in either group. In terms of patient-reported outcomes, both the AASCs and placebo groups showed symptom reductions, particularly in dry mouth and sticky saliva. In the AASCs group, dry mouth symptoms decreased by 13.6 units, while sticky saliva reduced by 14.8 units. In comparison, the placebo group had reductions of 7.7 units for dry mouth and 9.3 units for sticky saliva. However, these reductions did not lead to a statistically significant difference between the groups in terms of patient-reported symptoms.

**Figure 8:**
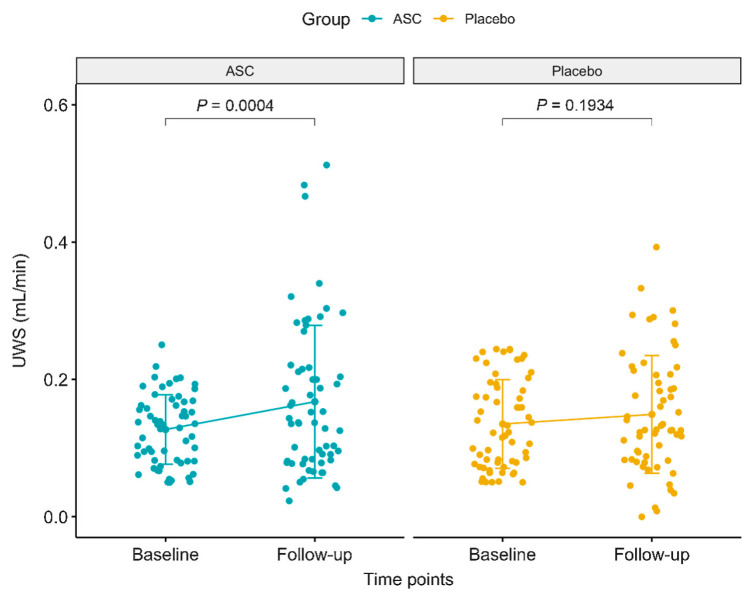
Unstimulated salivary flow rate. (Reprinted without modifications from Jakbsen et al. [23]).

Regarding safety, there were no treatment-related serious adverse events (SAEs), but nine patients in the AASCs group experienced temporary swelling of the submandibular glands, which resolved within 3 weeks. Additionally, 31% of patients in the AASCs group developed de novo human leukocyte antigen (HLA) antibodies, which may have influenced the overall efficacy, as those with new HLA antibodies tended to have a lesser increase in salivary flow rates (Figure 8).

In conclusion, ASC therapy improved salivary function and reduced xerostomia symptoms, though the improvements were not significantly greater than those observed in the placebo group.

#### 3.3.3. Treatment of Type 1 Diabetes Mellitus

A study by Araujo et al. [24] evaluated the safety and potential efficacy of adipose tissue-derived allogeneic stem cells (AASCs) combined with vitamin D in patients recently diagnosed with type 1 diabetes (T1D). A total of 13 patients participated: eight received a single dose of AASCs (1 × 10^6^ cells/kg) along with vitamin D (2000 IU/day for three months), while five followed standard insulin therapy.

The results showed that adverse events were generally mild and transient. Among the eight patients who received AASCs, 100% reported transient headaches, 88% reported mild local reactions, 50% experienced tachycardia, and 13% reported abdominal cramps. Four patients (50%) developed superficial local thrombophlebitis, and two (25%) experienced transient “floaters”. One case of central retinal vein occlusion was reported at three months, with complete resolution.

In terms of pancreatic function, C-peptide (CP) levels were significantly higher in the AASC group at the beginning of the study (225.90 ± 92.91 ng/mL) compared to the control group (110.55 ± 29.72 ng/mL, *p* = 0.02). One month after the intervention, CP levels were 250.22 ± 129.49 ng/mL in the AASC group versus 127.35 ± 18.31 ng/mL in the control group (*p* = 0.03). At three months, CP levels were 211.20 ± 100.42 ng/mL in the AASC group versus 106.05 ± 47.25 ng/mL in the control group, although the difference was no longer statistically significant (*p* = 0.06).

**Figure 9:**
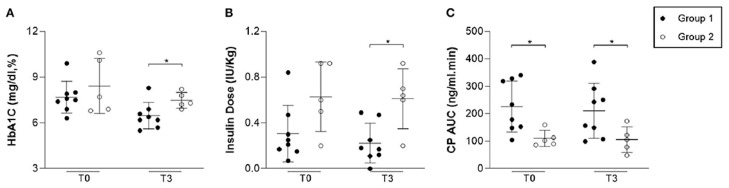
(**A**) HbA1C levels measured before (T0) and three months after (T3) ASCs infusion. Group 1 showed significantly lower HbA1C levels compared to Group 2 (*p* = 0.03). (**B**) Insulin dose requirements before (T0) and three months after (T3) ASCs infusion. Group 1 required a significantly lower insulin dose than Group 2 (*p* = 0.01). (**C**) C-Peptide AUC measured before (T0) and three months after (T3) ASCs infusion. There was no significant difference in C-Peptide AUC between Group 1 and Group 2 (*p* = 0.06). The asterisks in the figure indicate statistically significant differences between the groups. (Reprinted without modifications from Araujo et al. [24]).

Glycemic control improved in the AASC group. Insulin requirements decreased significantly, from 0.31 ± 0.24 IU/kg at baseline to 0.22 ± 0.17 IU/kg at three months, compared to an increase in the control group (from 0.62 ± 0.30 IU/kg to 0.61 ± 0.26 IU/kg, *p* = 0.01). HbA1c levels also reduced to 6.47 ± 0.86% in the AASC group compared to 7.48 ± 0.52% in the control group at three months (*p* = 0.03). Two patients in the AASC group became temporarily insulin-independent (for 4 and 8 weeks), and 100% of the patients in this group were in the “honeymoon phase” at three months, compared to 0% in the control group (*p* = 0.01). (Figure 9).

This study demonstrates that the use of allogeneic stem cells derived from adipose tissue, combined with vitamin D, is associated with transient side effects, improved glycemic control, and a significant reduction in insulin requirements in newly diagnosed T1D patients. These results suggest promising therapeutic potential, although larger-scale studies with extended follow-up are needed to confirm these findings.

#### 3.3.4. Conclusions

While short-term symptomatic relief (tear and salivary flow, OSDI scores) appears reproducible, variations in dosing, delivery route and concurrent therapies introduce substantial heterogeneity. Longer-term data and head-to-head comparisons are needed to clarify durability and optimal protocols.

### 3.4. Lungs Diseases

#### 3.4.1. Community-Acquired Bacterial Pneumonia

A study conducted by Reijnders et al. [25] included 82 patients, divided equally between those receiving two intravenous infusions of Cx611 (AASCs) and a placebo group, all of whom suffered from severe community-acquired bacterial pneumonia requiring mechanical ventilation and/or vasopressor support. The use of Cx611 demonstrated a generally favorable safety profile and a measurable impact on host response dynamics. Baseline characteristics were well-balanced between the groups, with a similar mean age (60.9 ± 11.3 years for Cx611 and 63.4 ± 10.4 years for placebo) and nearly identical proportions of male patients (65.9% vs. 63.4%).

After 14 days, follow-up retention was high, with 85.4% of Cx611 patients (35/41) and 92.7% of placebo patients (38/41) still enrolled. Clinically, key indicators—including thromboembolic event rates (17.1% for Cx611 vs. 19.5% for placebo), median ICU stay duration (13 [6–29] days vs. 11 [6–19] days), hospital stay (20 [12–44] days vs. 19 [14–36] days) and 28-day mortality (19.5% vs. 14.6%)—were comparable between the two groups. This clinical stability underscores that the addition of Cx611 did not introduce significant risks, a critical consideration for such a severe condition.

**Figure 10:**
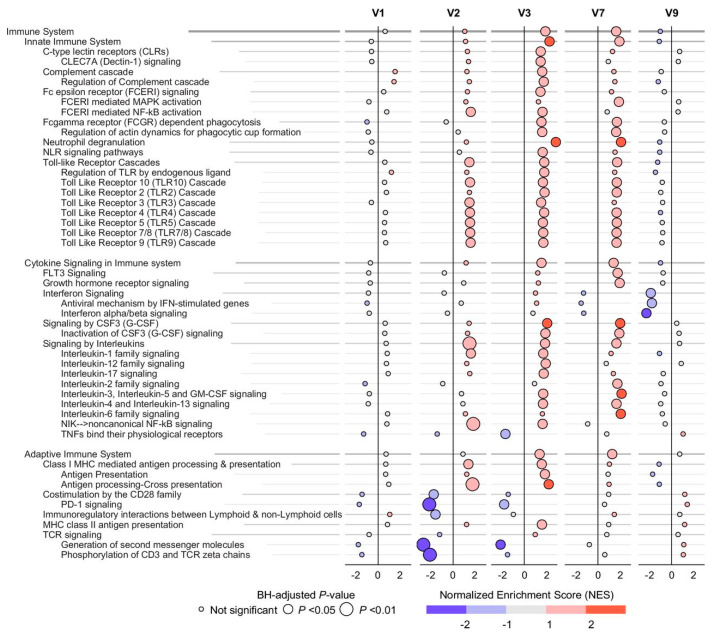
Significant immune system pathways identified through gene set enrichment analysis of the blood transcriptome. This bubble plot illustrates how Cx611 treatment influences transcriptional pathways related to the immune system (based on the Reactome knowledgebase) at each time point following initiation of Cx611 or placebo treatment. To account for random baseline differences in gene expression between groups, the differences at each time point are calculated using interaction terms between “Cx611” and “time point” in linear mixed models, with the screening (SCR) time point (before treatment) serving as the reference. Hence, these differences represent how gene expression in one group differs from the other at each subsequent time point relative to their pre-treatment levels. The differences are expressed as Normalized Enrichment Scores (NES), depicted by color intensity: red indicates higher expression in the Cx611 group, blue indicates lower expression, and grey signifies minimal difference. The bubble size corresponds to the Benjamini–Hochberg (BH)-adjusted p value for that pathway. Abbreviations: CLEC7A, C-type lectin domain family 7 member A; Fc, fragment crystallizable region; FLT3, fms-related receptor tyrosine kinase 3; G-CSF, granulocyte colony-stimulating factor; GM-CSF, granulocyte-macrophage colony-stimulating factor; IFN, interferon; MAPK, mitogen-activated protein kinase; MHC, major histocompatibility complex; NF-κB, nuclear factor kappa-light-chain-enhancer of activated B cells; NIK, NF-κB-inducing kinase; NLR, nucleotide-binding domain leucine-rich repeat containing receptor; PD-1, programmed death 1; TCR, T cell receptor; TNF, tumor necrosis factor. (Reprinted without modifications from Reijnders et al. [25]).

Biologically, an analysis of 29 plasma biomarkers revealed a transient modulation of pathways related to the endothelium, coagulation and immunity. For instance, temporary increases in factors such as von Willebrand factor, prothrombin fragments 1 + 2 and D-dimers might reflect a physiological rebalancing aimed at optimizing tissue perfusion and vascular repair without an associated increase in complications. Additionally, a modest rise in TNF levels (*p* = 0.030) suggests an adaptation of immune response, hinting at the potential of Cx611 to promote a beneficial reconfiguration of the host defense system (Figure 10).

Laterre et al. [26] conducted a study involving 83 patients, of which 42 received allogeneic adipose tissue-derived mesenchymal stem cells (Cx611), and 41 were administered a placebo. The data clearly highlight several advantages of Cx611 compared to the placebo, particularly regarding tolerability. Only one patient treated with Cx611 (1/42) experienced hypernatremia, whereas six patients in the placebo group (6/41) reported this condition. Additionally, no anaphylactic reactions were observed in the Cx611 group, while one case occurred in the placebo group, underscoring the favorable safety profile of this cellular treatment.

Immunologically, among the 35 patients in the Cx611 group who initially lacked anti-HLA antibodies, only three (approximately 8.6%) developed them by day 14, and two of these patients saw their antibodies disappear by day 90, suggesting a transient and non-harmful immune response. This immunological stability indicates good integration of Cx611 without long-lasting sensitization.

Clinically, several patients treated with Cx611 exhibited a gradual improvement in their condition over the follow-up period. Furthermore, hypersensitivity reactions and thromboembolic events were favorable under the introduced treatment (Cx611: n = 9; placebo: n = 12). The mean number of ventilator-free days over 28 days was 12.2 for Cx611 compared to 15.4 for the placebo. Hypersensitivity reactions and thromboembolic events were less frequent with Cx611 (nine cases) than with placebo (12 cases), indicating overall better safety with the cellular treatment.

#### 3.4.2. Severe COVID-19 Pneumonia

A study by Zhu et al. [27] included seven patients with severe COVID-19 pneumonia (four men and three women, median age: 57 years [IQR: 43–70 years]). The median duration between symptom onset and hospitalization was 30 days (IQR: 15–40 days), and the administration of adipose-derived mesenchymal stem cell (AASC)-derived exosomes (AASCs) by inhalation began, on average, 54 days after symptom onset (IQR: 34–69 days). Each patient received a daily dose of 2.0 × 10^8^ exosomes for five consecutive days, resulting in a cumulative dose of 1.0 × 10^9^ exosomes per patient.

All patients tolerated the inhalation of AASCs well. No predefined adverse events (e.g., fever, respiratory distress, diarrhea, seizures) or clinical instability were observed during or immediately after nebulization. Vital parameters (temperature, heart rate, respiratory rate and oxygen saturation) remained stable throughout the five days of treatment.

**Figure 11:**
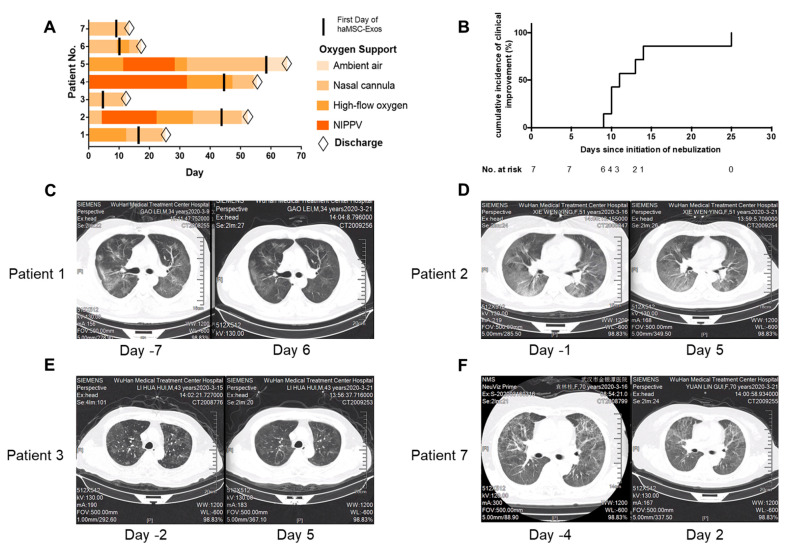
Clinical improvements compared to baseline (the first day of inhalation therapy) in individual patients participating in the MEXCOVID study. (**A**) Changes in each patient’s oxygen support requirements from baseline. (**B**) Cumulative incidence of clinical improvement starting from the initiation of nebulization treatment. (**C**–**F**) Differences in chest CT images before and after AASCs inhalation in COVID-19 patients. (Reprinted without modifications from Zhu et al. [27]).

Biologically, there was a trend toward an improvement in blood lymphocyte count (median: 1.61 × 10^9^/L before treatment vs. 1.78 × 10^9^/L after treatment). Most patients showed a reduction in inflammatory markers, such as CRP (decreased in six out of seven patients), IL-6 (decreased in five out of seven patients) and LDH (decreased in six out of seven patients). Liver and kidney functions remained stable, with no significant increases in ALT or creatinine levels (Figure 11).

Radiological evaluation demonstrated improvement in pulmonary lesions on CT scans by day seven, with a reduction in the lung severity score (median: 51 points before treatment vs. 40 points after treatment, *p* = 0.0559). Four out of seven patients exhibited more marked resolution of radiological abnormalities. Additionally, two patients on high-flow oxygen therapy were able to transition to standard oxygen therapy following the treatment.

#### 3.4.3. Conclusions

The two controlled trials in severe pneumonia demonstrate good tolerability and biologically plausible modulation of host-response pathways, but they were under-powered to detect mortality benefits. Future multicenter phase III studies should focus on hard clinical endpoints and stratify for baseline immune status.

### 3.5. Acute Ischemic Stroke

A phase IIa clinical trial published by De Celis-Ruiz et al. [28] included 19 patients to evaluate the safety of intravenous administration of adipose-derived mesenchymal stem cells (AASCs) in patients with acute ischemic stroke. Of these 19 patients, six were excluded: two due to technical issues related to cell manufacturing and four for meeting exclusion criteria after randomization. The final sample consisted of 13 patients, divided into two groups: four received AASCs treatment, and nine received a placebo.

The median time from symptom onset to treatment administration was slightly longer in the AASCs group at 13 days (IQR: 13–13.75) compared to 12 days (IQR: 10–13) in the placebo group (*p* = 0.028). Baseline demographic and clinical characteristics were comparable between the two groups, with a median age of 78 years in the AASCs group and 76 years in the placebo group.

Over the 24-month follow-up period, 124 adverse events (AEs) were reported, with 50 in the AASCs group and 74 in the placebo group (*p* = 0.074). None of these events were attributed to the AASCs treatment. Additionally, 11 serious adverse events (SAEs) were documented: two in the AASCs group and nine in the placebo group, including one death in the placebo group during the first week, attributed to multiorgan failure.

In terms of efficacy parameters, no statistically significant differences were observed between the groups regarding functional scores (modified Rankin Scale, mRS) or infarct volume. However, a positive trend was noted in NIHSS scores, with a median score of 3 (IQR: 3–6.75) in the AASCs group compared to 7 (IQR: 0–12.5) in the placebo group at 24 months.

**Figure 12:**
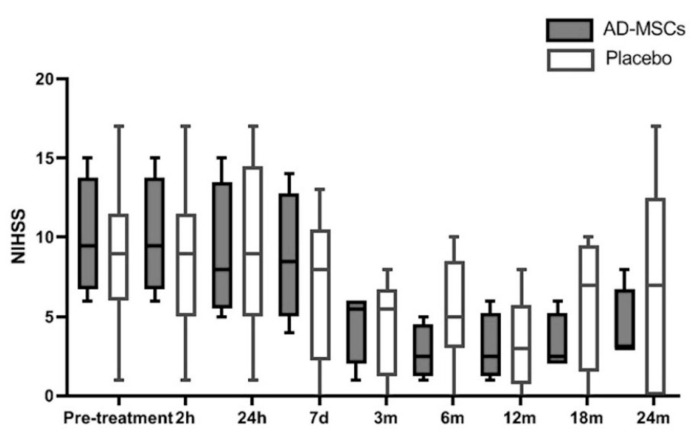
NIHSS scores observed over a 24-month follow-up period. AASCs: adipose tissue-derived mesenchymal stem cells; NIHSS: National Institutes of Health Stroke Scale. (Reprinted without modifications from De Celis-Ruiz et al. [28]).

These findings indicate that the intravenous administration of AASCs is safe and well-tolerated, while also highlighting a potential clinical benefit, particularly in terms of long-term neurological score improvements (Figure 12).

The trial proves feasibility and safety, but its tiny sample and late-treatment window mean efficacy remains speculative. A larger, earlier-intervention RCT is now indispensable before AASCs can be advocated for stroke recovery.

### 3.6. Kidney Diseases

Zheng et al. [29] aimed to evaluate the safety and tolerability of AASCs in patients with chronic kidney disease (CKD). Twelve eligible CKD patients with an estimated glomerular filtration rate (eGFR) of 15–44 mL/min/1.73 m^2^ received one dose of allogeneic stem cells (ELIXCYTE^®^, UnicoCell Biomed, Co. Ltd. Taipei 11494, Taiwan) intravenously in three groups: three low-dose (6.4 × 10^7^ cells in a total of 8 mL); three medium-dose (19.2 × 10^7^ cells in a total of 24 mL); and six high-dose (32.0 × 10^7^ cells in a total of 40 mL), and the groups were evaluated after 48 weeks.

**Figure 13:**
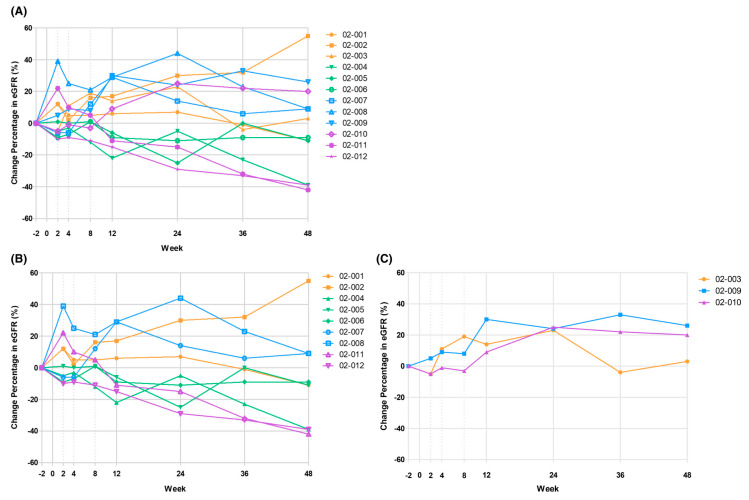
Changes in eGFR compared to baseline across 48 weeks in: (**A**): all participants who received treatment; (**B**): participants with baseline eGFR < 30 mL/min; (**C**) participants with baseline eGFR ≥30 mL/min. (Reprinted without modifications from Zheng et al. [29]).

The primary endpoint was the safety profile in terms of the incidence of adverse events and serious adverse events. An increase in eGFR was observed in seven of 12 subjects (58%) at week 24 and in six of 12 subjects (50%) at week 48. At week 24, an increase in eGFR of more than 20% was observed in all CKD patients with an initial eGFR ≧ 30 mL/min/1.73 m^2^, compared with only two subjects in the group with an initial eGFR < 30 mL/min/1.73 m^2^. No significant reduction in proteinuria was observed in all subjects. This study demonstrated that a single intravenous dose of ELIXCYTE^®^ was well tolerated in patients with moderate-to-severe CKD, and that its preliminary efficacy warranted further studies (Figure 13).

Single-arm results validate tolerability, yet heterogeneous eGFR rises could simply reflect natural fluctuation. Only a randomized, controlled phase-II study with renal histology and hard renal endpoints can test true disease-modifying potential.

### 3.7. Spinal Muscular Atrophy (SMA1)

Mohseni et al. [30] conducted a clinical trial among 10 SMA1 pediatric patients using AASCs (mean age at disease onset, 3 ± 1 months). Electrodiagnosis revealed a reduction in action potentials in both groups. There were no serious adverse events in the intervention group. All patients left the hospital without immediate post-injection complications. There was no significant difference in the number of hospitalizations or days on ventilator between the cell therapy and control groups. Assessments at 3 to 48 months after stem cell transplantation showed no acceleration of disease progression. However, except for one patient in the intervention group, all died of respiratory problems, with an average life expectancy of 11.17 months for the intervention group vs. 8.52 months for the control group. Ballard scores assessing the patients’ condition were slightly higher in the intervention group than in the control group, but without a significant difference. Median motor nerve response amplitude was higher in the intervention group. This trend held for the ulnar and tibial nerves, but not for the peroneal nerve.

After the third stem cell injection, a significant difference was observed only for the tibial nerve. One patient in the intervention group survived up to 48 months, showing improvement in thoracic muscle movements, allowing breathing without intubation and following a normal growth pattern. AASCs therapy is safe and shows promising efficacy for the treatment of SMAI. An early intervention could improve its efficacy. The study suggests the use of this treatment assessment to monitor treatment efficacy.

No infusion-related toxicity emerged, but the modest survival gain in this small cohort is inconclusive. Controlled trials that pair AASCs with today’s standard SMA therapeutics are needed to clarify any additive benefit.

### 3.8. Osteoarthritis

The injection of mesenchymal stromal cells has been proposed as an innovative treatment for osteoarthritis of the knee. These cells, particularly those derived from adipose tissue, are considered a preferable choice in regenerative medicine due to their availability as ready-to-use products.

Sadri et al. [31] studied three patients with osteoarthritis of the knee. Each received an intraarticular injection of one hundred million AASCs into each affected knee. Follow-up lasted 6 months, during which time clinical outcomes, MRI and serum inflammatory biomarkers were assessed. The primary objective was to verify the safety and feasibility of injecting AASCs during 6 months of follow-up. No serious adverse events were reported. Analysis of secondary outcomes, such as the Visual Analogue Scale, the Western Ontario and McMaster Universities Osteoarthritis (WOMAC) Index and the Knee Osteoarthritis Outcome Score, showed improvement in all patients. The decrease in the WOMAC score, considered as an indicator of improvement, initially occurred with a steep slope and fell from 54, 43 and 53 to 16, 19 and 8 for patient 1, patient 2 and patient 3, respectively, after 3 months, and to decrease until the end of the 6-month follow-up. 

**Figure 14:**
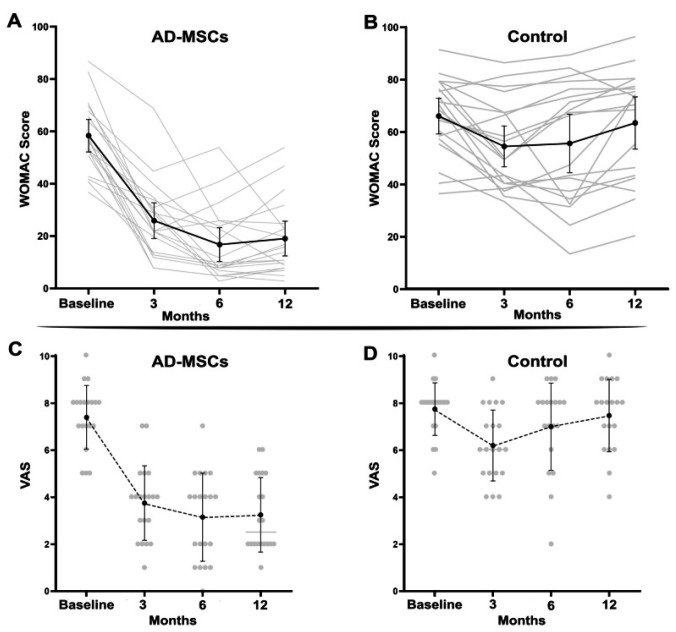
(**A**) WOMAC scores in the AD-MSCs group: a marked and sustained decrease in scores, indicating significant improvement in pain and function following treatment. (**B**) WOMAC scores in the control group: no significant change in scores, reflecting the absence of clinical improvement. (**C**) VAS scores in the AD-MSCs group: a rapid and persistent reduction in pain levels, as shown by lower VAS scores throughout follow-up. (**D**) VAS scores in the control group: pain levels remain stable or slightly increase, with no meaningful improvement observed. The WOMAC refers to the Western Ontario and McMaster Universities Osteoarthritis Index, while the VAS represents the Visual Analog Scale. The data points are shown as mean values, with error bars reflecting the 95% confidence intervals. Statistical analysis was performed using a one-way repeated measures ANOVA. (Reprinted without modifications from Sadri et al. [31]).

Changes in cartilage volume and thickness, joint effusion and subchondral edema were identified by comparing MRI results before and 6 months after AASCs treatment. AASCs injection not only increased cartilage volume, but also reduced subchondral edema and joint effusion. In addition, the decrease in serum levels of cartilage oligomeric matrix protein and hyaluronic acid suggests a reduction in cartilage degeneration. Quantification of interleukin-10 and interleukin-6 levels showed immunomodulation after cell injection (Figure 14).

In a randomized, triple-blind, phase II study, Sadri et al. [32] included 40 patients with knee osteoarthritis. Of these, 20 received an intra-articular injection of 100 × 10^6^ AASCs, while 20 received a placebo (saline solution). Four patients were excluded during the study, leaving 36 participants for the final analysis (18 in each group). The mean age was 52.8 ± 7.5 years in the AASCs group and 56.1 ± 7.2 years in the placebo group.

No serious adverse events (SAEs) were observed. Two patients in the AASCs group reported swelling and local pain at the injection site, which resolved within 2 to 3 days without intervention. Laboratory parameters, such as CRP and erythrocyte sedimentation rate, remained normal.

The clinical outcomes following treatment with allogeneic adipose-derived mesenchymal stem cells (AASCs) demonstrated significant and sustained improvements across multiple validated patient questionnaires. Specifically, results from the WOMAC questionnaire indicated a substantial improvement in the AASCs group, with mean scores decreasing notably from 58.35 ± 13.25 at baseline to 16.75 ± 13.81 after 6 months and maintaining at 19.05 ± 14.12 at 12 months, reflecting a reduction of more than 70% at 6 months. Conversely, patients in the placebo group showed minimal variation, with scores marginally declining from 65.42 ± 14.63 at baseline to 63.47 ± 20.68 after 12 months.

Pain assessments via the Visual Analog Scale (VAS) corroborated these findings, revealing a marked decrease in reported pain intensity in the AASCs-treated patients, from an initial score of 7.40 ± 1.35 down to 3.15 ± 1.87 at 6 months, and stabilizing at 3.25 ± 1.58 by the 12-month mark. The placebo group, however, experienced negligible change, maintaining pain scores around baseline levels (7.73 ± 1.14 initially and 7.47 ± 1.54 after 12 months).

Evaluations using the KOOS score, indicative of knee functionality, further highlighted the effectiveness of the AASCs intervention. In the treatment group, the mean KOOS score markedly improved from 28.30 ± 14.90 at baseline to 69.15 ± 12.22 at 6 months, with a slight decline to 63.75 ± 15.40 at 12 months. In stark contrast, scores in the placebo group remained essentially unchanged, evolving minimally from 22.05 ± 9.57 at baseline to 23.84 ± 15.09 at the end of the study period.

**Figure 15:**
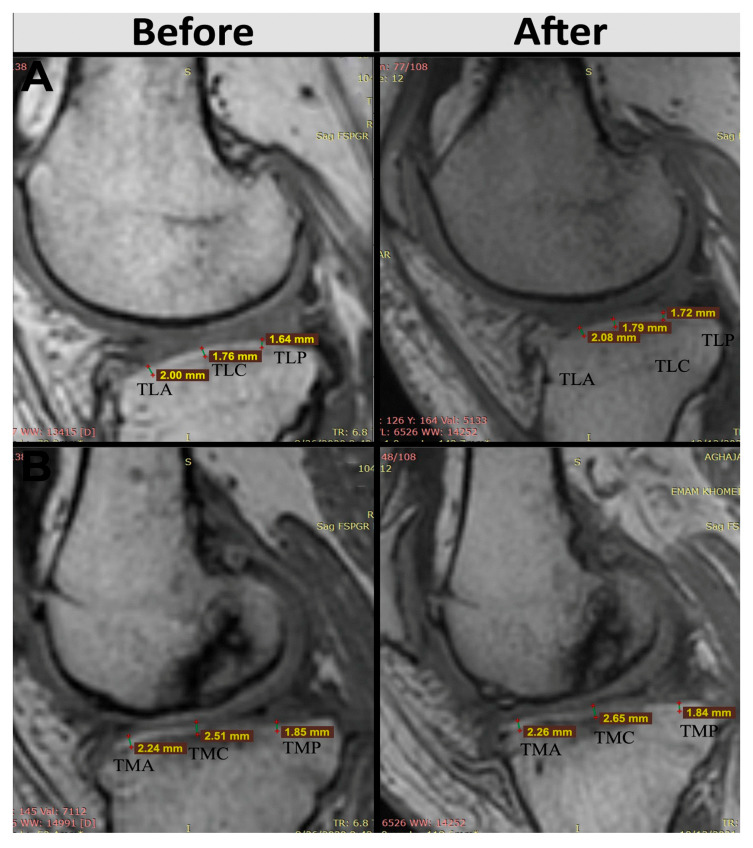
MRI assessment of the tibial condyle cartilage before and 48 weeks after AASCs injection. (**A**) The lateral view shows increased cartilage thickness in the anterior, posterior and central regions of the tibial condyle. (**B**) The medial view highlights an enhancement in cartilage thickness across the anterior, posterior and central areas of the tibial condyle. MRI: magnetic resonance imaging; TLA: tibia lateral anterior; TLC: tibia lateral central; TLP: tibia lateral posterior; TMA: tibia medial anterior; TMC: tibia medial central; TMP: tibia medial posterior; AASCs: adipose-derived mesenchymal stromal cells. (Reprinted without modifications from Sadri et al. [32]).

In addition to these clinical improvements, structural changes in articular cartilage thickness were objectively assessed at predefined anatomical landmarks on the tibia and femur. Patients treated with AASCs exhibited increased cartilage thickness in the medial anterior tibial region (TMA), rising from 1.86 ± 0.53 mm to 1.98 ± 0.56 mm at 12 months, and in the medial posterior tibial region (TMP), which increased from 2.01 ± 0.29 mm to 2.07 ± 0.26 mm. Conversely, placebo recipients experienced a reduction in TMA cartilage thickness, dropping from 1.49 ± 0.88 mm to 1.37 ± 0.81 mm, with TMP thickness remaining essentially unchanged (1.81 ± 0.25 mm to 1.78 ± 0.18 mm) (Figure 15).

These combined clinical and imaging findings clearly illustrate the therapeutic potential of AASCs for improving both symptomatic and structural outcomes in patients.

Chen et al. [33] studied 11 patients with knee osteoarthritis received intra-articular injections of AASCs at two different doses: a low dose of 6.7 × 10^6^ cells (n = 5) and a high dose of 4 × 10^7^ cells (n = 6). Over a follow-up period of 12 weeks, significant improvements were observed, particularly in the high-dose group.

The results showed that the WOMAC total score, which evaluates pain, stiffness and physical function, dropped substantially in the high-dose group compared to baseline. At week 12, the WOMAC pain scores decreased by an average of 7.7 points, while stiffness scores improved by 3.1 points, and physical function scores saw an improvement of 14.3 points. In contrast, the low-dose group showed only moderate improvements, with smaller reductions in WOMAC scores across all categories.

VAS scores for pain also reflected similar trends. Patients in the high-dose group reported a significant reduction in pain, with an average decrease of 4.5 points on the VAS scale by week 12. The low-dose group, while also showing improvement, had a less pronounced reduction in pain, averaging around 2.1 points of improvement.

Additionally, MRI imaging conducted at 12 weeks post-injection demonstrated structural changes in the high-dose group. These patients showed increased cartilage volume, along with reduced subchondral edema and joint effusion, suggesting not only symptomatic relief but potential structural improvements in the knee joint. The low-dose group did not exhibit significant changes in MRI results.

**Figure 16:**
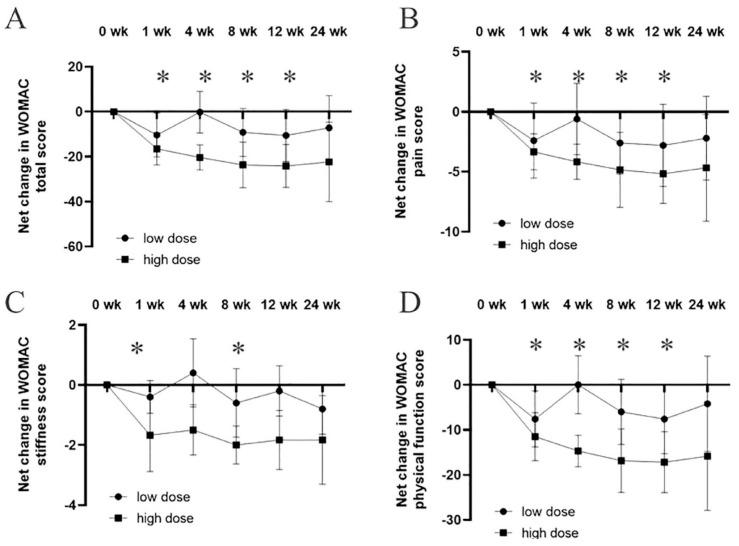
Changes in WOMAC scores from baseline to 24 weeks following intra-articular injection of GXCPC1 (AASCs). (**A**) Total WOMAC score, (**B**) WOMAC pain score, (**C**) WOMAC stiffness score, and (**D**) WOMAC physical function score. *: Indicates a significant difference observed in the high-dose group. (Reprinted without modifications from Chen et al. [33]).

Overall, 73% of patients in the high-dose group reported a marked improvement in their symptoms, while only 40% of patients in the low-dose group experienced comparable benefits. These results indicate that higher doses of AASCs lead to greater clinical improvements in pain relief, joint function, and even cartilage regeneration in patients with knee osteoarthritis (Figure 16).

Lu et al. [34] conducted a study involving 22 patients with bilateral knee osteoarthritis, divided into three groups based on the administered dose of AASCs: low dose (1 × 10^7^ cells, seven patients), medium dose (2 × 10^7^ cells, eight patients) and high dose (5 × 10^7^ cells, seven patients). Among these participants, 19 were women, with a mean age of 57.9 years and a mean BMI of 26.3 kg/m^2^. Three patients withdrew from the study for reasons unrelated to the treatment. Regarding safety, 19 patients (86.4%) reported at least one adverse event, the most common being localized pain (81.8%) and knee swelling (27.3%). These adverse effects were transient, resolving within three days, and no serious adverse events attributable to the treatment were observed.

Clinically, significant improvements were observed at 48 weeks. The total WOMAC score, assessing pain, stiffness and joint function, decreased by 23.71 points in the low-dose group, 16.50 points in the medium-dose group, and 10.71 points in the high-dose group, reflecting an improved quality of life for patients. Additionally, pain scores measured by the Visual Analog Scale (VAS) decreased by 2.19 points for the left knee in the low-dose group, 2.25 points in the medium-dose group, and 1.36 points in the high-dose group. Overall quality of life, measured by the SF-36 questionnaire, improved by 22.71 points in the low-dose group, 12.63 points in the medium-dose group, and 10.57 points in the high-dose group.

MRI analyses revealed an increase in cartilage volume in the low-dose group, with an average gain of 54.58 mm^3^ in total cartilage volume and 39.69 mm^3^ in tibial cartilage. Conversely, reductions in cartilage volume were observed in the medium- and high-dose groups. The WORMS score, assessing changes in joint structures, showed a decrease of 0.36 points in the left knee and 0.86 points in the right knee in the low-dose group, while the other groups exhibited minimal significant changes.

Pain, function and cartilage signals are encouraging, yet placebo and imaging-bias cannot be ruled out because two studies lacked sham controls. A multicenter, sham-controlled phase-III RCT is therefore essential to confirm clinical value.

### 3.9. Patients with Severely Refractory Graft-Versus-Host Disease (GvHD)

Prasad et al. [35] conducted a study on the use of human AASCs in the treatment of acute GvHD (aGvHD). This study investigated the use of Prochymal^TM^ (Osiris Therapeutics), a prefabricated formulation of human AASCs in the treatment of aGVHD in children. In total, 12 children (10 boys, two girls; age, 0.4–15 years) suffering from treatment-resistant grades III and IV aGVHD were treated on a compassionate basis at five transplant centers between July 2005 and June 2007. These children, presenting mainly with severe gastrointestinal symptoms and liver and/or skin damage for one-half, were refractory to steroids and several other immunosuppressive therapies. AASCs were administered intravenously at variable doses twice weekly for four weeks, followed by additional weekly doses for partial or mixed responses.

Results showed that 58% of patients had a complete response, 17% a partial response, and 25% a mixed response. Complete resolution of gastrointestinal symptoms was observed in 75% of patients. Survival at 100 days after the start of treatment was 58%, with 42% of patients still alive after a median follow-up of 611 days. No acute infusion-related or other toxicities were observed, indicating that multiple infusions of AASCs are well tolerated and appear safe in children.

**Figure 17:**
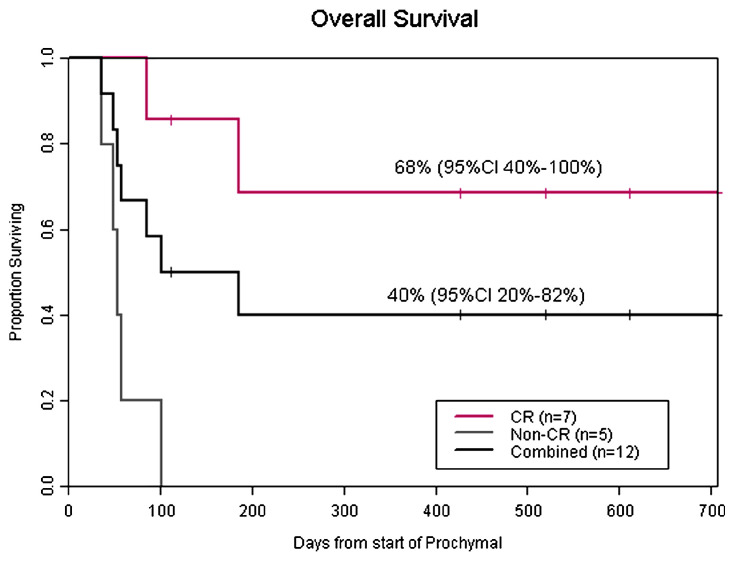
Kaplan–Meier estimates illustrating the probability of 2-year overall survival based on graft-versus-host disease response subsequent to Prochymal^TM^ (Osiris Therapeutics) therapy. Among the participants, seven patients achieved a complete response (CR) to Prochymal^TM^, while the remaining five patients (non-CR) exhibited partial or mixed responses. Surviving patients were monitored for a median duration of 611 days, ranging from 427 to 1111 days. (Reprinted without modifications from Prasad et al. [35]).

The compassionate-use series reports a notable 58% complete-response rate in a highly refractory pediatric population, yet the absence of a control arm and concomitant therapies confound causal inference. Randomized, placebo-controlled trials currently in progress will be crucial to validate these findings (Figure 17).

## 4. Discussion

Adipose-derived mesenchymal stem cells are moving decisively from experimental proof-of-concept to a clinically credible, platform-level therapy. The most coherent way to understand the accumulating evidence is to follow the biological continuum that these cells engage—tissue repair, immune recalibration and vascular support—rather than to revisit every individual indication in isolation. Taken together, >950 treated patients across phase I and II trials demonstrate a strikingly uniform safety profile: adverse events are predominantly mild and self-limiting, no malignant transformation has been observed, and clinically relevant allo-sensitization remains undocumented even when third-party donors are used. This consolidated tolerability allows investigators to interrogate efficacy signals with a confidence that few first-in-class cell products enjoy.

From a regenerative standpoint, AASCs accelerate re-epithelialization of thermal injuries and chronic wounds, remodel hypertrophic scars, and restore pigmentation effects that reflect both multilineage differentiation capacity and a paracrine “recruitment” of resident progenitors. Similar mechanisms appear to underpin the structural restitution observed in cartilage, renal parenchyma and even the post-ischemic brain. The phase II trial by De Celis-Ruiz et al. [28], in which intravenously infused AASCs delivered within two weeks of an acute ischemic stroke yielded a median 24-month NIHSS score of 3 vs. 7 for placebo, illustrates the magnitude of functional recovery that may be attainable when treatment coincides with the subacute repair window. Equally compelling is the durable restoration of salivary flow in patients with radiation-induced xerostomia and the normalization of tear-film stability in experimental dry-eye models—findings that confirm the ability of AASCs to regenerate highly specialized glandular tissues and hint at broader applications in exocrine dysfunction.

Immunologically, AASCs secrete IL-10, TGF-β, prostaglandin E_2_ and indoleamine-2,3-dioxygenase, shifting T-cell polarization toward a regulatory phenotype without the blanket immunosuppression characteristic of corticosteroids or calcineurin inhibitors. Refractory perianal fistulae in Crohn’s disease, which often survive multiple courses of anti-TNF therapy, have been closed durably by local implantation of allogeneic AASCs. In pediatric acute graft-versus-host disease, systemic infusion dampened cytokine storm activity while preserving overall immune competence—a qualitative advantage in a population already burdened by infection risk. Early open-label studies in psoriasis similarly report reductions in plaque severity linked to normalization of the Th17/Treg ratio, and vitamin-D-primed AASCs administered at the onset of type 1 diabetes have reduced exogenous insulin requirements, consistent with partial preservation of β-cell mass. These data collectively argue that AASCs do more than suppress inflammation; they recalibrate it in a way that preserves host defense and promotes tissue restitution.

The third unifying thread is vascular support. AASCs, and the exosomes they shed, release VEGF, HGF and endothelial-protective microRNAs such as miR-126 and miR-21. This trophic cocktail promotes angiogenesis in diabetic foot ulcers, improves renal perfusion markers in early chronic kidney disease, and appears to stabilize hemodynamics in severe community-acquired bacterial pneumonia treated with the allogeneic product Cx611. Nebulized AASC exosomes in COVID-19 pneumonia have proven feasible, safe, and potentially advantageous for distal airway deposition; most recipients exhibited radiographic improvement, mild lymphocyte rebound, and falling CRP and IL-6 levels. Although the sample was small and confounded by concomitant antivirals and corticosteroids, these observations suggest that local delivery can leverage the vascular and immunological properties of AASCs without the uncertainties of systemic cell trafficking.

Despite these converging mechanistic signals, the field remains constrained by small, often single-center trials, heterogeneous manufacturing protocols, and reliance on surrogate endpoints. Detailed isolation protocols covering harvest site, liposuction settings, collagenase type and exposure time, wash-and-filtration steps, and the first hours of culture are rarely disclosed, even though these parameters decisively shape AASC phenotype and secretome. Without consistent reporting of those procedures, together with passage number and cryopreservation conditions, comparing cell potency across trials or deriving a reliable dose–response curve becomes virtually impossible. Moreover, the timing of administration is inconsistent: some stroke trials infuse cells within two weeks, whereas others wait up to three months; COVID-19 studies often treat patients already entering spontaneous recovery. Such variability almost certainly masks potential benefit in under-treated windows and exaggerates it in late, self-limiting phases. To move beyond proof-of-concept, phase III trials must therefore align on potency assays, target windows and clinically meaningful primary endpoints, ideally event-driven measures such as ulcer closure at 12 weeks, fistula recurrence at one year, or independence from disease-modifying drugs.

Our own synthesis of the evidence suggests a pragmatic clinical hierarchy. Complex perianal fistulae and diabetic foot ulcers are closest to regulatory approval: the burden of disease is high, the proposed mechanism is local and anatomically contained, and placebo-controlled data already show meaningful benefit. By contrast, severe viral pneumonias and spinal muscular atrophy should remain exploratory. In COVID-19, the natural history of the disease, the multiplicity of concomitant treatments, and evolving viral variants make it difficult to isolate a cell-specific effect; SMA, meanwhile, now competes with highly effective gene-replacement therapy, raising the bar for added value. Stroke, osteoarthritis and chronic kidney disease occupy a middle ground where robust phase III data could tip the balance if they demonstrate functional gains that persist beyond two years and translate into quality-adjusted life-year (QALY) savings.

Ethical and economic considerations are equally pivotal. Allogeneic sourcing may circumvent the morbidity of autologous liposuction, but it requires rock-solid donor consent, traceability and virological safety testing, as well as long-term surveillance for allo-immunity. Strategies such as closed bioreactor expansion, pooled donor batches, and point-of-care cryobanking could help reach this target, but only if regulators agree on harmonized, risk-based standards that reward demonstrable potency rather than mere compliance with legacy protocols.

Looking forward, three priorities emerge. First, rigorous multicenter phase III trials with biomarker-anchored patient stratification are indispensable if AASCs are to secure licensure. Second, mechanistic biomarkers—single-cell transcriptomics of injected cells, longitudinal exosomal cargo profiling, and host cytokine fingerprints—should be incorporated prospectively to test causal hypotheses and to refine dosing. Third, rational combinations merit exploration: vitamin D for metabolic priming in autoimmunity, collagen or hyaluronic acid scaffolds for orthopedic lesions, and targeted biologics to synergize with paracrine signals in inflammatory bowel disease.

In summary, AASCs have crossed the safety threshold and arrived at the efficacy inflection point. Their transition from experimental promise to routine clinical practice will not be driven by yet another small phase II signal but by the field’s collective ability to deliver harmonized, event-based phase III evidence that withstands economic and ethical scrutiny. Should that standard be met, AASCs may inaugurate a new therapeutic era in which cell products are deployed not as last-line salvage therapies but as mechanism-guided, first-intention interventions across regenerative medicine, immunology and vascular science.

## 5. Conclusions

Adipose-derived mesenchymal stem cells have progressed from experimental curiosity to a credible therapeutic platform. Clinical experience involving more than one thousand recipients shows a reproducibly benign safety profile, with adverse events generally mild, no malignant transformation observed, and not clinically significant allo-sensitization despite the routine use of allogeneic donors. Early efficacy signals converge on three mutually reinforcing mechanisms: promotion of tissue regeneration, modulation of dysregulated immune responses, and provision of a trophic, angiogenic environment that stabilizes microvascular integrity. This mechanistic coherence argues for viewing AASCs as a unified regenerative technology rather than a collection of indication-specific interventions.

The next decisive milestone is the generation of phase-three evidence produced under harmonized, Good-Manufacturing-Practice conditions. Trials must incorporate rigorous randomization, masking, and clinically meaningful, event-based end points, while release criteria should quantify viability, immunophenotype, and secretome activity to ensure batch-to-batch comparability. Prospectively embedded biomarker programs—spanning single-cell transcriptomics, exosomal micro-RNA profiling, and host cytokine signatures—will refine patient selection, clarify mechanism, and guide dosing. Long-term pharmacovigilance through internationally linked registries is essential for detecting late immunological or oncogenic sequelae and for verifying durability of benefit.

Successful translation will also depend on a transparent ethical architecture. Impeccable donor consent, rigorous lot traceability, inclusive trial recruitment and equitable global access are non-negotiable. At the same time, manufacturing workflows must be refined—through closed-bioreactor expansion, pooled donor banking, and seamless cryochain logistics—to deliver products at a cost that health-care systems can sustain. Strategic convergence with complementary modalities, such as vitamin D priming, targeted biologics or biomaterial scaffolds, offers additional opportunities to enhance potency without eroding feasibility.

To summarize, AASCs have satisfied the foundational requirements of safety and biological plausibility and have generated coherent, mechanism-anchored signs of clinical benefit. Their transition from promise to practice now rests on the field’s capacity to deliver harmonized, event-driven phase-three data within a robust ethical and regulatory framework. Achieving this will not merely expand the therapeutic arsenal; it will mark the arrival of cell-based, mechanism-guided medicine as an integral component of mainstream clinical care.

## Figures and Tables

**Table 1:** Table lists each study by first author (year) and summarizes its design. For every trial, we indicate whether allocation was randomized, whether any form of masking (blinding) was used, and whether a concurrent control/comparator group was included. Where applicable, the type of comparator (placebo, active vehicle, fibrin-gel only, inert placebo, non-treatment observation or untreated controls) or the blinding level (single-, double- or triple-blind).

Authors (Years)	Study Design	Randomized	Blinded	Controlled	Risk and Bias Assessment (Rob 2.0/ROBINS-1)	References
Gentile (2023)	Prospective Cohort Study (Observational; OCEBM Level II)	No	No	Yes (comparative groups)	High	[14]
Moon et al. (2019)	Multicenter, Parallel-Group Clinical Trial	Yes, Computer-Generated, Center-Stratified, Permuted-Block Randomization (1:1)	Yes—single-blind	Yes—comparator-controlled	Moderate	[15]
Mrozikiewicz-Rakowska et al. (2023)	Prospective, Parallel-Group Clinical Trial	No	No	Yes—comparator (fibrin-gel only) arm	Moderate	[16]
Bajouri et al. (2023)	Phase I Single-Arm, Open-Label Clinical Trial	No	No	No	Moderate	[17]
Panés et al. (2016)	Phase III Multicenter, Parallel-Group Clinical Trial	Yes, Central, Computer-Generated Allocation List (1:1), Stratified by Baseline Concomitant Therapy	Yes—double-blind	Yes—placebo-controlled	Low	[18]
Garcia-Olmo et al. (2022)	Phase III Controlled Clinical Trial	Yes, Central, Computer-Generated Allocation List (1:1), Stratified by Baseline Concomitant Therapy	Yes—double-blind	Yes—placebo-controlled	Low	[19]
Maciel Gutiérrez et al. (2021)	Phase I Prospective, Single-Arm Clinical Trial	No	No	No	Moderate	[20]
Møller-Hansen et al. (2024)	Phase I Clinical Trial	Yes, Central, Computer-Generated, Simple 1:1 Randomization	Yes—double-blind	Yes—active-comparator (vehicle) and non-treatment observation groups	Moderate	[21]
Lynggaard et al. (2022)	Prospective Phase Ib Single-Arm Safety Study	No	No	No	High	[22]
Jakobsen et al. (2024)	Phase II Clinical Trial	Yes, Computer-Generated, Block Randomization (1:1)	No	Yes—comparator arm received an inert placebo	Low	[23]
Araujo et al. (2020)	Prospective Phase II Pilot Trial	No	No	Yes— comparator arm received insulin therapy alone	Moderate	[24]
Reijnders et al. (2024)	Phase Ib/IIa Clinical Trial	Yes, Central, Computer-Generated, Simple 1:1 Randomization	Yes—double-blind	Yes—placebo-controlled	Low	[25]
Laterre et al. (2024)	Phase Ib/IIa Clinical Trial	Yes, Central, Computer-Generated, Simple 1:1 Randomization	Yes—double-blind	Yes—placebo-controlled	Low	[26]
Zhu et al. (2022)	Phase IIa Single-Arm, Interventional Pilot Study	No	No	No	High	[27]
De Celis-Ruiz et al. (2022)	Phase IIa Single-Center, Randomized Pilot Clinical Trial	Yes, Computer-Generated, Simple 1:1 Parallel-Group Randomization,	Yes—double-blind	Yes—placebo-controlled	Low	[28]
Zheng et al. (2022]	Phase I Single-Center, Dose-Escalation Feasibility/Safety Trial	No	No	No	High	[29]
Mohseni et al. (2022)	Phase I Dose-Escalation Clinical Trial	Yes, Computer-Generated, Simple 1:1 Open-Label Randomization	No	Yes—with an untreated control group	Moderate	[30]
Sadri et al. (2022)	Phase I Single-Arm Feasibility/Safety Study	No	No	No	High	[31]
Sadri et al. (2023)	Phase II Clinical Trial	Yes, Stratified, Permuted-Block Randomization (1:1)	Triple-blinded	Yes—placebo-controlled	Low	[32]
Chen et al. (2024)	Phase I Dose-Escalation Pilot Study	No	No	No	Moderate	[33]
Lu et al. (2020)	Phase I Dose-Ranging Clinical Trial	Yes, Computer-Generated, Permuted-Block Randomization (1:1)	Yes—double-blind	No	Moderate	[34]
Prasad et al. (2011)	Multicenter Compassionate-Use (Expanded-Access) Clinical Series	No	No	No	High	[35]

**Table 2:** Summary of clinical response to AASCs by indication.

Subject of Study	Number of Studies	Number of Patients in Study	Patients Who Received AASCs	Patients Who Received a Benefit from AASCs	Patients Who Did Not Benefit from AASCs	Predominant Design/Randomization	Overall Risk Assessment	References
Skin lesions	4	190	103	93 (90.3%)	10 (9.7%)	2 RCTs (computer blocks) + 2 single arm	Moderate	[14,15,16,17]
Crohn’s disease	3	271	151	88 (58.3%)	63 (41.7%)	2 RCTs (web randomizer) + 1 Phase I	Low	[18,19,20]
Glandular dysfunctions	4	197	102	51 (50%)	51 (50%)	3 RCTs (sealed envelopes) + 1 single-arm	Moderate	[21,22,23,24]
Lugs Diseases	3	172	90	62 (68.9%)	28 (31.1%)	2 RCTs + 1 open-label	Moderate	[25,26,27]
Stroke	1	13	4	4 (100%)	0 (0%)	Phase IIa RCT (computer blocks)	Low	[28]
Renal dysfunction	1	12	12	6 (50%)	6 (50%)	Single-arm dose-escalation	High	[29]
Neurodegenerative pathology	1	10	5	5 (100%)	0 (0%)	Single-arm	High	[30]
Osteoarthritis	4	76	56	50 (89.3%)	6 (10.7%)	1 triple-blind RCT + 3 open-label	Moderate	[31,32,33,34]
Acute graft-versus- host disease	1	12	12	9 (75%)	3 (25%)	Compassionate-use series	High	[35]
Total	22	953	535	368	167			

## Data Availability

Not applicable.

## References

[1] Brignier A.C., Gewirtz A.M. (2010). Embryonic and adult stem cell therapy. J. Allergy Clin. Immunol..

[2] Monti M., Perotti C., Del Fante C., Cervio M., Redi C.A. (2012). Stem cells: Sources and therapies. Biol. Res..

[3] Dehghanifard A., Shahjahani M., Soleimani M., Saki N. (2013). The emerging role of mesenchymal stem cells in tissue engineering. Int. J. Hematol. Oncol. Stem Cell Res..

[4] Laloze J., Fiévet L., Desmoulière A. (2021). Adipose-Derived Mesenchymal Stromal Cells in Regenerative Medicine: State of Play, Current Clinical Trials, and Future Prospects. Adv. Wound Care.

[5] Bacakova L., Zarubova J., Travnickova M., Musilkova J., Pajorova J., Slepicka P., Molitor M. (2018). Stem cells: Their source, potency and use in regenerative therapies with focus on adipose-derived stem cells–a review. Biotechnol. Adv..

[6] Zuk P.A., Zhu M., Ashjian P., De Ugarte D.A., Huang J.I., Mizuno H., Hedrick M.H. (2002). Human Adipose Tissue Is a Source of Multipotent Stem Cells. Mol. Biol. Cell.

[7] Carmeliet P. (2005). Angiogenesis in life, disease and medicine. Nature.

[8] Andrae J., Gallini R., Betsholtz C. (2008). Role of platelet-derived growth factors in physiology and medicine. Genes Dev..

[9] Cheng Y.S., Yen H.H., Chang C.Y., Lien W.C., Huang S.H., Lee S.S., Wang H.M.D. (2022). Adipose-Derived Stem Cell-Incubated HA-Rich Sponge Matrix Implant Modulates Oxidative Stress to Enhance VEGF and TGF-β Secretions for Extracellular Matrix Reconstruction In Vivo. Oxidative Med. Cell. Longev..

[10] Mehrabani D., Babazadeh M., Tanideh N., Zare S., Hoseinzadeh S., Torabinejad S., Koohi-Hosseinabadi O. (2015). The Healing Effect of Adipose-Derived Mesenchymal Stem Cells in Full-thickness Femoral Articular Cartilage Defects of Rabbit. Int. J. Organ Transplant. Med..

[11] Schäffler A., Büchler C. (2007). Concise Review: Adipose Tissue-Derived Stromal Cells—Basic and Clinical Implications for Novel Cell-Based Therapies. Stem Cells.

[12] Ciervo Y., Ning K., Jun X., Shaw P.J., Mead R.J. (2017). Advances, challenges and future directions for stem cell therapy in amyotrophic lateral sclerosis. Mol. Neurodegener..

[13] Shukla L., Yuan Y., Shayan R., Greening D.W., Karnezis T. (2020). Fat Therapeutics: The Clinical Capacity of Adipose-Derived Stem Cells and Exosomes for Human Disease and Tissue Regeneration. Front. Pharmacol..

[14] Gentile P. (2023). Lipofilling Enriched with Adipose-Derived Mesenchymal Stem Cells Improves Soft Tissue Deformities and Reduces Scar Pigmentation: Clinical and Instrumental Evaluation in Plastic Surgery. Aesthetic Plast. Surg..

[15] Moon K.C., Suh H.S., Kim K.B., Han S.K., Young K.W., Lee J.W., Kim M.H. (2019). Potential of Allogeneic Adipose-Derived Stem Cell–Hydrogel Complex for Treating Diabetic Foot Ulcers. Diabetes.

[16] Mrozikiewicz-Rakowska B., Szabłowska-Gadomska I., Cysewski D., Rudziński S., Płoski R., Gasperowicz P., Lewandowska-Szumiel M. (2023). Allogenic Adipose-Derived Stem Cells in Diabetic Foot Ulcer Treatment: Clinical Effectiveness, Safety, Survival in the Wound Site, and Proteomic Impact. Int. J. Mol. Sci..

[17] Bajouri A., Dayani D., Sharghi A.T., Karimi S., Niknejadi M., Bidgoli K.M., Vosough M. (2023). Subcutaneous Injection of Allogeneic Adipose-Derived Mesenchymal Stromal Cells in Psoriasis Plaques: Clinical Trial Phase I. Cell J..

[18] Panés J., García-Olmo D., Van Assche G., Colombel J.F., Reinisch W., Baumgart D.C., Danese S. (2016). Expanded allogeneic adipose-derived mesenchymal stem cells (Cx601) for complex perianal fistulas in Crohn’s disease: A phase 3 randomised, double-blind controlled trial. Lancet.

[19] Garcia-Olmo D., Gilaberte I., Binek M., Lindner D., Selvaggi F., Spinelli A., Panés J. (2022). Follow-up Study to Evaluate the Long-term Safety and Efficacy of Darvadstrocel (Mesenchymal Stem Cell Treatment) in Patients With Perianal Fistulizing Crohn’s Disease: ADMIRE-CD Phase 3 Randomized Controlled Trial. Dis. Colon Rectum.

[20] Maciel Gutiérrez V.M., Guillen S.G.G., Flores M.W.C., Pérez J.A.V., Rendón F.M.A., García F.S.H., Torres G.Á.G. (2021). Safety of Allogeneic Adipose Tissue-Derived Mesenchymal Stem Cells for the Treatment of Complex Perianal Fistulas Not Associated With Crohn’s Disease: A Phase I Clinical Trial. Dis. Colon Rectum.

[21] Møller-Hansen M., Larsen A.C., Wiencke A.K., Terslev L., Siersma V., Andersen T.T., Heegaard S. (2024). Allogeneic mesenchymal stem cell therapy for dry eye disease in patients with Sjögren’s syndrome: A randomized clinical trial. Ocul. Surf..

[22] Lynggaard C.D., Grønhøj C., Christensen R., Fischer-Nielsen A., Melchiors J., Specht L., von Buchwald C. (2022). Intraglandular Off-the-Shelf Allogeneic Mesenchymal Stem Cell Treatment in Patients with Radiation-Induced Xerostomia: A Safety Study (MESRIX-II). Stem Cells Transl. Med..

[23] Jakobsen K.K., Carlander A.L.F., Todsen T., Melchiors J., Paaske N., Østergaard Madsen A.K., von Buchwald C. (2024). Mesenchymal Stem/Stromal Cell Therapy for Radiation-Induced Xerostomia in Previous Head and Neck Cancer Patients: A Phase II Randomized, Placebo-Controlled Trial. Clin. Cancer Res..

[24] Araujo D.B., Dantas J.R., Silva K.R., Souto D.L., Pereira M.D.F.C., Moreira J.P., Rodacki M. (2020). Allogenic Adipose Tissue-Derived Stromal/Stem Cells and Vitamin D Supplementation in Patients with Recent-Onset Type 1 Diabetes Mellitus: A 3-Month Follow-Up Pilot Study. Front. Immunol..

[25] Reijnders T.D., Laterre P.F., François B., García M.S., van Engelen T.S., Sie D., van der Poll T. (2024). Effect of mesenchymal stem cells on the host response in severe community-acquired pneumonia. Thorax.

[26] Laterre P.F., García M.S., van der Poll T., Wittebole X., Martínez-Sagasti F., Hernandez G., SEPCELL Study, Group (2024). The safety and efficacy of stem cells for the treatment of severe community-acquired bacterial pneumonia: A randomized clinical trial. J. Crit. Care.

[27] Zhu Y.G., Shi M.M., Monsel A., Dai C.X., Dong X., Shen H., Qu J.M. (2022). Nebulized exosomes derived from allogenic adipose tissue mesenchymal stromal cells in patients with severe COVID-19: A pilot study. Stem Cell Res. Ther..

[28] De Celis-Ruiz E., Fuentes B., Alonso De Leciñana M., Gutiérrez-Fernández M., Borobia A.M., Gutiérrez-Zúñiga R., Díez-Tejedor E. (2022). Final Results of Allogeneic Adipose Tissue–Derived Mesenchymal Stem Cells in Acute Ischemic Stroke (AMASCIS): A Phase II, Randomized, Double-Blind, Placebo-Controlled, Single-Center, Pilot Clinical Trial. Cell Transplant..

[29] Zheng C.M., Chiu I.J., Chen Y.W., Hsu Y.H., Hung L.Y., Wu M.Y., Wu M.S. (2022). Allogeneic adipose tissue-derived stem cells ELIXCYTE^®^ in chronic kidney disease: A phase I study assessing safety and clinical feasibility. J. Cell. Mol. Med..

[30] Mohseni R., Hamidieh A.A., Shoae-Hassani A., Ghahvechi-Akbari M., Majma A., Mohammadi M., Ashrafi M.R. (2022). An open-label phase 1 clinical trial of the allogeneic side population adipose-derived mesenchymal stem cells in SMA type 1 patients. Neurol. Sci..

[31] Sadri B., Tamimi A., Nouraein S., Bagheri Fard A., Mohammadi J., Mohammadpour M., Vosough M. (2022). Clinical and laboratory findings following transplantation of allogeneic adipose-derived mesenchymal stromal cells in knee osteoarthritis, a brief report. Connect. Tissue Res..

[32] Sadri B., Hassanzadeh M., Bagherifard A., Mohammadi J., Alikhani M., Moeinabadi-Bidgoli K., Vosough M. (2023). Cartilage regeneration and inflammation modulation in knee osteoarthritis following injection of allogeneic adipose-derived mesenchymal stromal cells: A phase II, triple-blinded, placebo controlled, randomized trial. Stem Cell Res. Ther..

[33] Chen C.F., Chen Y.C., Fu Y.S., Tsai S.W., Wu P.K., Chen C.M., Chuang M.H. (2024). Safety and Tolerability of Intra-Articular Injection of Adipose-Derived Mesenchymal Stem Cells GXCPC1 in 11 Subjects with Knee Osteoarthritis: A Nonrandomized Pilot Study Without a Control Arm. Cell Transplant..

[34] Lu L., Dai C., Du H., Li S., Ye P., Zhang L., Bao C. (2020). Intra-Articular Injections of Allogeneic Human Adipose-Derived Mesenchymal Progenitor Cells in Patients with Symptomatic Bilateral Knee Osteoarthritis: A Phase I pilot study. Regen. Med..

[35] Prasad V.K., Lucas K.G., Kleiner G.I., Talano J.A.M., Jacobsohn D., Broadwater G., Kurtzberg J. (2011). Efficacy and Safety of Ex Vivo Cultured Adult Human Mesenchymal Stem Cells (Prochymal^TM^) in Pediatric Patients with Severe Refractory Acute Graft-Versus-Host Disease in a Compassionate Use Study. Biol. Blood Marrow Transplant..

